# Dissemination of Tn*916*-Related Integrative and Conjugative Elements in Streptococcus pneumoniae Occurs by Transformation and Homologous Recombination in Nasopharyngeal Biofilms

**DOI:** 10.1128/spectrum.03759-22

**Published:** 2023-03-13

**Authors:** Brenda S. Antezana, Sarah Lohsen, Xueqing Wu, Jorge E. Vidal, Yih-Ling Tzeng, David S. Stephens

**Affiliations:** a Microbiology and Molecular Genetics Program, Graduate Division of Biological and Biomedical Sciences, Emory University Laney Graduate School, Atlanta, Georgia, USA; b Department of Medicine, Division of Infectious Diseases, Emory University School of Medicine, Atlanta, Georgia, USA; c Department of Infectious Diseases, Sir Run Run Shaw Hospital, Zhejiang University College of Medicine, Hangzhou, China; d Department of Cell and Molecular Biology, University of Mississippi Medical Center, Jackson, Mississippi, USA; University of Pittsburgh School of Medicine

**Keywords:** *Streptococcus pneumoniae*, antibiotic resistance, biofilm, integrative and conjugative element, transformation

## Abstract

Multidrug resistance in Streptococcus pneumoniae (or pneumococcus) continues to be a global challenge. An important class of antibiotic resistance determinants disseminating in *S. pneumoniae* are >20-kb Tn*916*-related integrative and conjugative elements (ICEs), such as Tn*2009*, Tn*6002*, and Tn*2010*. Although conjugation has been implicated as the transfer mechanism for ICEs in several bacteria, including *S. pneumoniae*, the molecular basis for widespread dissemination of pneumococcal Tn*916*-related ICEs remains to be fully elucidated. We found that Tn*2009* acquisition was not detectable via *in vitro* transformation nor conjugative mating with donor GA16833, yielding a transfer frequency of <10^−7^. GA16833 Tn*2009* conjugative gene expression was not significantly induced, and ICE circular intermediate formation was not detected in biofilms. Consistently, Tn*2009* transfer efficiency in biofilms was not affected by deletion of the ICE conjugative gene *ftsK.* However, GA16833 Tn*2009* transfer occurred efficiently at a recombination frequency (rF) of 10^−4^ in dual-strain biofilms formed in a human nasopharyngeal cell bioreactor. DNase I addition and deletions of the early competence gene *comE* or transformation apparatus genes *comEA* and *comEC* in the D39 recipient strain prevented Tn*2009* acquisition (rF of <10^−7^). Genome sequencing and single nucleotide polymorphism analyses of independent recombinants of recipient genotype identified ~33- to ~55-kb donor DNAs containing intact Tn*2009*, supporting homologous recombination. Additional pneumococcal donor and recipient combinations were demonstrated to efficiently transfer Tn*916*-related ICEs at a rF of 10^−4^ in the biofilms. Tn*916*-related ICEs horizontally disseminate at high frequency in human nasopharyngeal *S. pneumoniae* biofilms by transformation and homologous recombination of >30-kb DNA fragments into the pneumococcal genome.

**IMPORTANCE** The World Health Organization has designated Streptococcus pneumoniae as a priority pathogen for research and development of new drug treatments due to extensive multidrug resistance. Multiple strains of *S. pneumoniae* colonize and form mixed biofilms in the human nasopharynx, which could enable exchange of antibiotic resistance determinants. Tn*916*-related integrative and conjugative elements (ICEs) are largely responsible for the widespread presence of macrolide and tetracycline resistance in *S. pneumoniae*. Utilizing a system that simulates colonization of donor and recipient *S. pneumoniae* strains in the human nasopharynx, efficient transfer of Tn*916*-related ICEs occurred in human nasopharyngeal biofilms, in contrast to *in vitro* conditions of planktonic cells with exogenous DNA. This high-frequency Tn*916*-related ICE transfer between *S. pneumoniae* strains in biofilms was due to transformation and homologous recombination, not conjugation. Understanding the molecular mechanism for dissemination of Tn*916*-related ICEs can facilitate the design of new strategies to combat antibiotic resistance.

## INTRODUCTION

Streptococcus pneumoniae (or pneumococcus) is a Gram-positive bacterial human pathogen and inhabitant of the human nasopharynx. Colonization with pneumococci occurs during early childhood, with carriage rates ranging from 20 to 93.4% and persisting into adulthood at lower levels (1–3). *S. pneumoniae* may remain as a commensal in the nasopharynx, spread to other mucosal sites, and cause otitis media or pneumonia, or it may invade the bloodstream, resulting in bacteremia or meningitis (4). Individuals at risk for invasive pneumococcal disease are children less than 2 years of age, those over 65 years of age, and immunocompromised individuals with other underlying chronic conditions (5). Although new, effective pneumococcal conjugate vaccines are available, invasive pneumococcal disease still poses as a global public health concern. In 2017, the World Health Organization (WHO) recognized *S. pneumoniae* as a priority pathogen due to extensive multidrug resistance (6). The widespread use of antibiotics, such as macrolides for treatment of community-acquired pneumonia and other upper respiratory infections, has led to marked increases in *S. pneumoniae* antibiotic resistance (7, 8). The horizontal transfer of resistance determinants followed by the selection of resistant strains is the major driver for the development of antibiotic resistance in *S. pneumoniae*, yet the molecular mechanisms of resistance determinant dissemination have not been clearly defined.

Large, mobile genetic elements known as integrative and conjugative elements (ICEs) of the Tn*916* family are responsible for tetracycline and macrolide resistance in *S. pneumoniae*. Tn*916*-related ICEs that have been widely identified in *S. pneumoniae* include Tn*2009* (23.5 kb), Tn*6002* (20.8 kb), and Tn*2010* (26.3 kb) (9–13). Although prototype Tn*916* (18.0 kb) and other classes of ICEs are disseminated via conjugation in several Gram-positive species, such as Bacillus subtilis, Enterococcus faecalis, Clostridium difficile, and many streptococcal species (14–16), the role of conjugation as an efficient horizontal exchange process for Tn*916*-related ICEs in *S. pneumoniae* is unclear. While conjugation of *S. pneumoniae* Tn*6002* into Streptococcus pyogenes has been demonstrated by mating experiments at the low frequency of 10^−8^, Tn*2009* and Tn*2010* did not conjugate into *S. pneumoniae* (10, 11). Another common horizontal genetic exchange strategy is transformation as *S. pneumoniae* naturally becomes competent for extracellular DNA uptake during growth (17). Transformation has long been associated with *S. pneumoniae* genome evolution (18, 19), particularly for acquisition of point mutations and small ~1- to ~5-kb determinants conferring antibiotic resistance.

To investigate the molecular mechanism for horizontal dissemination of Tn*916*-related ICEs among *S. pneumoniae* strains, we utilized a bioreactor system consisting of a dual-strain pneumococcal biofilm on human nasopharyngeal cells (20) with Tn*2009* as a model. Gene expression, mutation studies in key conjugative and transformation genes, ICE circular intermediate quantification, genome sequencing, and single nucleotide polymorphism (SNP) analyses revealed that transformation and homologous recombination are responsible for efficient horizontal transfer of *S. pneumoniae* Tn*2009*. These mechanistic observations were applicable to additional Tn*916*-related ICEs and *S. pneumoniae* strains.

## RESULTS

### Classic *in vitro* transformation failed to demonstrate acquisition of Tn*916*-related ICEs by *S. pneumoniae*.

*S. pneumoniae* is naturally competent for extracellular DNA (eDNA) uptake. Classic *in vitro* transformation assays were used to examine uptake of genomic DNA carrying ICEs. Planktonic cells of D39^Str^ or TIGR4, treated with the cognate synthetic competence-stimulating peptide (CSP) to initiate competence development, were incubated with various amounts of genomic DNA. Control reactions for competency demonstrated that D39^Str^ acquired trimethoprim (Tmp) resistance (*folA*/I100L) (21) at a recombination frequency (rF) of 4.08 × 10^−5^ ± 4.33 × 10^−5^ per μg DNA, whereas TIGR4 obtained streptomycin (Str) resistance (*rpsL*/K56T) (22) at a rF of 1.03 × 10^−5^ ± 7.88 × 10^−6^ per μg DNA. However, *in vitro* transformation of D39^Str^ under analogous conditions with genomic DNA harboring Tn*2009* (23.5 kb) or Tn*2010* (26.3 kb) yielded no recombinants with tetracycline (Tet) resistance, and the rFs were <1.13 × 10^−7^ ± 1.04 × 10^−7^ and <2.68 × 10^−8^ for Tn*2009* and Tn*2010*, respectively. Similarly, no Tet-resistant TIGR4 recombinants were recovered with genomic DNA carrying Tn*2009* (rF of <3.75 × 10^−8^ ± 3.63 × 10^−9^). Agarose gel electrophoresis confirmed that purified genomic DNA preparations contained fragments significantly larger than the ICEs (data not shown), and increasing the CSP concentrations from 100 ng/mL to 5000 ng/mL in *in vitro* transformations did not enhance recombination frequencies for uptake of Str resistance. Thus, classic *in vitro* transformation aided by exogenous CSP did not result in uptake of Tn*916-*related ICEs by D39 nor TIGR4.

### Efficient transfer of pneumococcal Tn*916*-related ICEs occurred in dual-strain biofilms formed on human nasopharyngeal cells.

A continuous flow bioreactor system, characterized by formation of dual-strain biofilms on a confluent monolayer of human pharyngeal Detroit 562 cells at 35°C has previously been shown to yield high rF for transfer of Str, Tmp, and Tet (*tetM*-mediated) resistance between D39 and TIGR4 (20). Thus, we investigated pneumococcal Tn*916*-related ICE dissemination using the bioreactor system with a 6-h incubation, which was previously found to be sufficient for efficient recombination. The ICE donor clinical isolates GA16833^Tet/Ery^ (serotype 19F with Tn*2009*) or GA47281^Tet/Ery^ (serotype 19F with Tn*2010*), were coinoculated in the bioreactor with designated recipient D39 (serotype 2) derivatives containing either Str (D39^Str^) or dual Str and Tmp (D39^Str/Tmp^) resistance.

**FIG 1:**
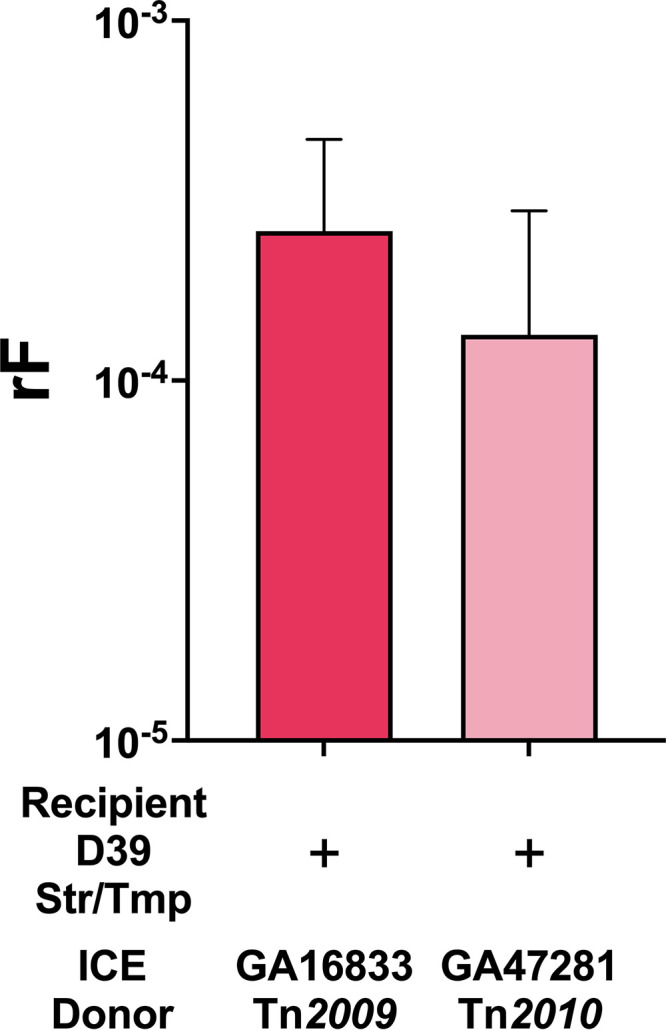
Efficient transfer of pneumococcal Tn*916*-related ICEs occurs in dual-strain nasopharyngeal biofilms. The wild-type recipient D39^Str/Tmp^ (serotype 2) and GA16833^Tet/Ery^ (with Tn*2009*, serotype 19F) or GA47281^Tet/Ery^ (with Tn*2010*, serotype 19F) were coinoculated in a bioreactor at 35°C on a confluent monolayer of human nasopharyngeal Detroit cells such that dual-strain biofilms formed. After a 6-h total incubation, recombination frequencies for D39 uptake of the tetracycline (Tet) resistance marker were calculated.

When GA16833^Tet/Ery^ served as the Tn*2009* donor, the rFs of Tet+Str resistance were 2.60 × 10^−4^ ± 2.08 × 10^−4^ for recipient D39^Str/Tmp^ (Fig. 1) and 1.34 × 10^−4^ ± 7.02 × 10^−5^ for recipient D39^Str^. The rF of 10^−4^ obtained in the bioreactor system represented an ~1,000-fold enhancement over the *in vitro* rF of <10^−7^ where no recombinants were recovered. Similarly, with coinoculation of the recipient D39^Str/Tmp^ and the Tn*2010* donor GA47281^Tet/Ery^, we obtained a rF of 1.34 × 10^−4^ ± 1.62 × 10^−4^ for Tet-resistant recombinants of D39^Str/Tmp^, once again demonstrating an ~1,000-fold enhancement over the *in vitro* transformation (Fig. 1). Additional selection combinations, Ery+Str, Tet+Ery+Str, and Ery+Str+Tmp, were also tested and yielded similar rFs (see Table S3 in the supplemental material). When Tet was used for recombinant selection, Tn*2009* and Tn*2010* yielded comparable rFs (10^−4^). However, when Ery was included in the selection, higher rF values were observed for Tn*2010* relative to those obtained with Tn*2009* (Table S3). This difference could be due to the constitutively expressed *ermB* on Tn*2010*, whereas Tn*2009* carries inducible Ery resistance on the macrolide efflux genetic assembly (Mega) element.

### Conjugation was not involved in the highly efficient transfer of Tn*916*-related ICEs (>20 kb) between pneumococci in bioreactor biofilms.

Transcriptional regulation of conjugation genes and the molecular mechanism resulting in Tn*916* conjugative transfer have been well characterized (23). Conjugation of Tn*916* is stimulated by tetracycline at the *tetM* promoter, which mediates antisense mRNA derepression of downstream regulatory genes (23, 24) and subsequently results in expression of the *xis* and *int* genes encoding essential enzymes for the excision of Tn*916* (23, 24) as well as the formation of the Tn*916* circular intermediate (CI). CI formation occurs via binding of coupling sequences on the 5′ and 3′ ends of the ICE (23, 25, 26). This physical association of the 5′ and 3′ ends of the circularized Tn*916* allows for upregulated transcription at the 3′ end of Tn*916* to extend through to the conjugative genes at the 5′ end, encoding the necessary conjugative machinery, such as the type IV secretion system. Subsequent passage of a single-stranded Tn*916* DNA from a donor to the recipient cell occurs (25), resulting in the integration of Tn*916* into the recipient genome via precise site-specific recombination (27). While conjugation of Tn*916* and other ICEs has been demonstrated in Gram-positive bacteria, we obtained several lines of evidence to exclude conjugation as the mechanism responsible for Tn*2009* dissemination among *S. pneumoniae* as described below.

### Conjugative gene expression in Tn*2009* was limited.

**FIG 2:**
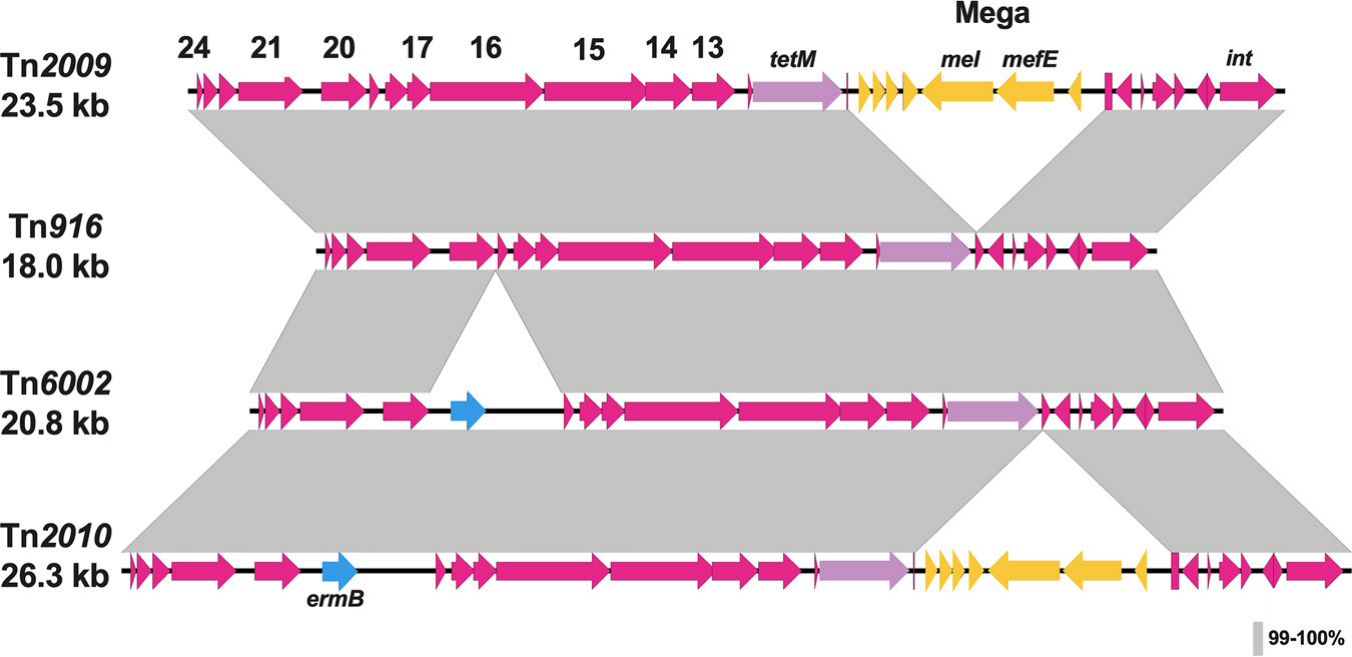
Sequence alignments of prototype Tn*916* with pneumococcal Tn*916*-related ICEs. EasyFig 2.2.2 was utilized to produce alignments of ICE elements using comparison files generated by BLASTN. Tetracycline resistance is conferred by the *tetM* gene (purple arrow) found in prototype Tn*916* as well as pneumococcal ICEs. Pneumococcal Tn*916*-related ICEs harbor macrolide resistance via the *ermB* element (blue arrows) found on Tn*6002* or Tn*2010* as well as the macrolide efflux genetic assembly (Mega) element (yellow arrows) on Tn*2009* or Tn*2010*. Conjugative genes shared by prototype Tn*916* and pneumococcal Tn*916*-related ICEs are denoted by magenta arrows.

We performed BLASTN analysis and demonstrated that all three pneumococcal Tn*916*-related ICEs contained genes encoding the excisionase, Xis, and the integrase, Int, which are required for Tn*916* excision and site-specific recombination (26, 28, 29). Tn*916*-related ICEs also contained open reading frames (ORFs) that had >99% sequence identity to the corresponding ORFs in Tn*916* involved in conjugative transfer (Fig. 2).

Expression of *xis* and *int* is coupled with tetracycline-inducible *tetM* transcription and subinhibitory concentrations of tetracycline, which result in enhanced conjugative transfer of Tn*916* in E. faecalis and B. subtilis (23, 30). We first compared the basal expression of conjugative genes in pneumococcal Tn*2009* with that of the prototype Tn*916* in a B. subtilis donor strain, CMJ253 (31). Expression of *tetM*, *int*, *orf20* (relaxase), and *ftsK* in Tn*2009* was significantly lower: *tetM* and *int* were expressed 19.7- and 32.4-fold lower, respectively, while *orf20* and *ftsK* were expressed about 300-fold lower than the corresponding genes in Tn*916*.

Tetracycline induces transcriptional upregulation of conjugative genes at the 5′ end of Tn*916* (23). To investigate if such a regulatory coupling was present in pneumococcal Tn*2009*, we grew the Tn*2009*-containing GA16833 strain (Tet MIC of 24 μg/mL) and the Tn*916*-containing CMJ253 strain in the presence or absence of 2.5 μg/mL tetracycline for 2.5 h (27). As expected for Tn*916*, there was a 4-fold induction of *tetM*, and similar levels of induction were detected for *orf20* and *ftsK* (Table 1). There was no induction of *int* expression in Tn*916* (Table 1), consistent with the observation by Celli et al. using Northern blotting (25). In contrast, while *tetM* expression in Tn*2009* was induced nearly 28-fold by a sublethal concentration of tetracycline, no concerted upregulation of conjugative genes was detected and only ~2.3- to ~3.7-fold changes were seen in *int*, *orf20*, and *ftsK* (Table 1) relative to no-tetracycline controls. Thus, unlike Tn*916*, conjugative gene expression was not coupled to *tetM* induction in Tn*2009*.

**TABLE 1:** Tetracycline-induced fold change in *tetM* and conjugative gene expression of Tn*916* and Tn*2009*

Strain	Expression (SD^*a*^) of			
	*tetM*	*int*	*orf20*	*ftsK*
CMJ253 (Tn*916*)	4.00 (0.071)	1.10 (0.053)	4.02 (0.62)	3.34 (0.095)
GA16833 (Tn*2009*)	28.18 (15.24)	2.25 (1.08)	2.79 (0.99)	3.72 (0.38)

a SD represents the standard deviation from two independent biological replicates.

We also investigated if conjugative gene expression in the Tn*2009* donor was induced in the dual-strain biofilm of recipient D39^Str^ and donor GA16833 *ΔcomCDE*::*cat*^Cm/Tet/Ery^ (BASP1), where Tn*2009* transfer was detected at high frequency. When normalized to broth cultures of the single donor strain BASP1 or a 1:1 mixture of donor and recipient strains, there was no induction of conjugative genes in biofilms, with a fold change of 1.13 or 1.04 for *int* and 0.28 or 0.94 for *orf20*, respectively. These data suggested that conjugative gene expression from pneumococcal Tn*2009* was minimal and not induced under the bioreactor biofilm conditions, and thus was unlikely to support efficient Tn*2009* transfer.

### There was no detectable circular intermediate formation of Tn*2009*.

Circular intermediate (CI) formation resulting from excision from the donor chromosome and circularization of Tn*916*, mediated by Xis and Int proteins, is a prerequisite for Tn*916* conjugative transfer and is induced by tetracycline (25, 26). The circular junction is the same between the prototype Tn*916* and Tn*2009*. Thus using quantitative PCR (qPCR), we quantified CIs derived from Tn*2009* in GA16833 and Tn*916* in CMJ253 with tetracycline induction. The CI copy number was normalized to the chromosomal copy number of *ftsK*. The positive control, B. subtilis donor CMJ253, produced a Tn*916* CI/chromosome ratio of 2.50 × 10^−2^ ± 1.42 × 10^−2^, while the *S. pneumoniae* donor GA16833 yielded no detectable Tn*2009* CIs, with a ratio of <2.48 × 10^−7^ ± 1.47 × 10^−7^.

**FIG 3:**
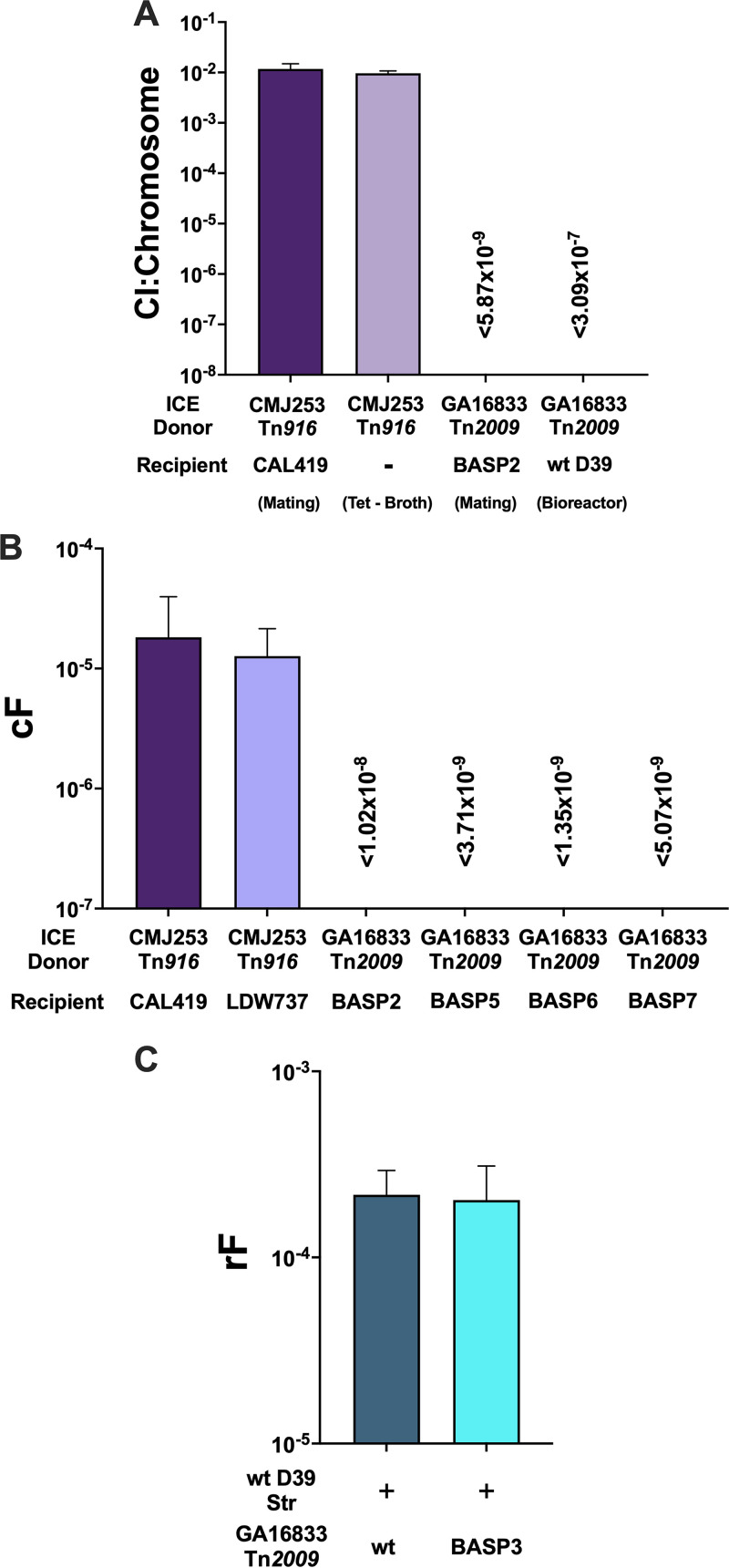
Pneumococcal Tn*2009* does not form circular intermediates (CIs) nor transfer by conjugation. (A) Circular intermediate quantification of 4-h mating reactions performed utilizing 10^8^ CFU of both the donor and recipient as well as bioreactor biofilms consisting of wild-type recipient D39^Str^ and wild-type donor GA16833^Tet/Ery^. The data represent the frequency of CI copy number normalized to copy number of *ftsK* that is present on Tn*2009* and Tn*916*. (B) Conjugation frequencies from mating reactions of 10^8^:10^9^ CFU of donor-recipient mixtures. (C) Wild-type recipient D39^Str^ was coinoculated with wild-type donor GA16833^Tet/Ery^ or BASP3 (GA16833 Δ*ftsK*) in the bioreactor. After a 6-h incubation, recombination frequencies for D39 uptake of Tet resistance were calculated.

We further examined CI production under two additional conditions (15, 32): mating assays used for conjugation and bioreactor biofilm reactions that yielded efficient Tn*2009* transfer. A positive control of donor CMJ253 broth culture in the absence of tetracycline produced a Tn*916* CI ratio of 9.66 × 10^−3^ ± 1.06 × 10^−3^, while the bioreactor samples did not yield detectable Tn*2009* CIs, with a ratio of <3.09 × 10^−7^ ± 1.56 × 10^−7^ (Fig. 3A). Mating assays of B. subtilis donor CMJ253 and recipient CAL419 resulted in a Tn*916* CI ratio of 1.17 × 10^−2^ ± 3.14 × 10^−3^, while mating reactions of *S. pneumoniae* donor GA16833 and recipient D39 *ΔcomE*::*cat*^Str^ (BASP2) did not yield detectable CIs (ratio of <5.87 × 10^−9^ ± 7.11 × 10^−9^) (Fig. 3A). These data confirmed the lack of pneumococcal Tn*2009* CI formation, either by tetracycline induction or under dual-strain conditions, such as mating reactions and bioreactor biofilms.

### Mating reactions did not support conjugative transfer of Tn*2009*.

Transfer of large mobile genetic elements via conjugation has been observed in mating experiments (15, 32). We reproduced this observation with mating experiments of B. subtilis Tn*916* donor CMJ253 and two B. subtilis recipients, LDW737 (27) and CAL419 (31). We obtained conjugation frequencies (cFs) of 1.27 × 10^−5^ ± 8.76 × 10^−6^ and 1.82 × 10^−5^ ± 2.14 × 10^−5^, respectively (Fig. 3B). However, mating between the *S. pneumoniae* Tn*2009* donor GA16833 and BASP2, an incompetent *S. pneumoniae* recipient used to eliminate the contribution of transformation, yielded no transconjugants, with an estimated cF of <1.02 × 10^−8^ ± 8.80 × 10^−9^ (Fig. 3B). Santoro et al. (15) had shown that conjugative transfer of Tn*5251* occurred in a strain-dependent manner in *S. pneumoniae*. Thus, three additional recipients were investigated: TIGR4 *ΔcomE*::*cat*^Tmp^ (BASP5), GA40410 *ΔcomE*::*cat*^Tmp^ (BASP6), and GA43265 *ΔcomE*::*cat*^Tmp^ (BASP7). Mating experiments with the donor GA16833 did not yield Tn*2009* Tet-resistant transconjugants, and the cFs were <3.71 × 10^−9^ ± 3.53 × 10^−9^, <1.35 × 10^−9^ ± 6.82 × 10^−10^, and <5.07 × 10^−9^ ± 4.57 × 10^−9^, respectively (Fig. 3B).

### Mutation of *ftsK* had no effect on the transfer of Tn*2009* in mixed nasopharyngeal biofilms.

During conjugation, Orf21 (FtsK) of Tn*916* serves as a coupling protein for translocating the DNA through the secretion system from the donor to recipient cell (33–35). Given the critical role of a coupling protein in the conjugative machinery, an *ftsK* mutant was created in the donor strain (GA16833 *ΔftsK*::*cat*^Tet/Ery^ [BASP3]). Coinoculation of donor BASP3 with recipient D39^Str^ in the bioreactor yielded a rF of ~10^−4^, similar to that obtained when wild-type GA16833 served as the Tn*2009* donor. Thus, disruption of conjugation via the *ftsK* mutation had no impact on the transfer of Tn*2009* (Fig. 3C), supporting that conjugation played no significant role in the highly efficient dissemination of Tn*2009* under the bioreactor biofilm conditions.

### Competence development and transformation machinery in the pneumococcal recipient were required for efficient ICE uptake in human nasopharyngeal biofilms.

**FIG 4:**
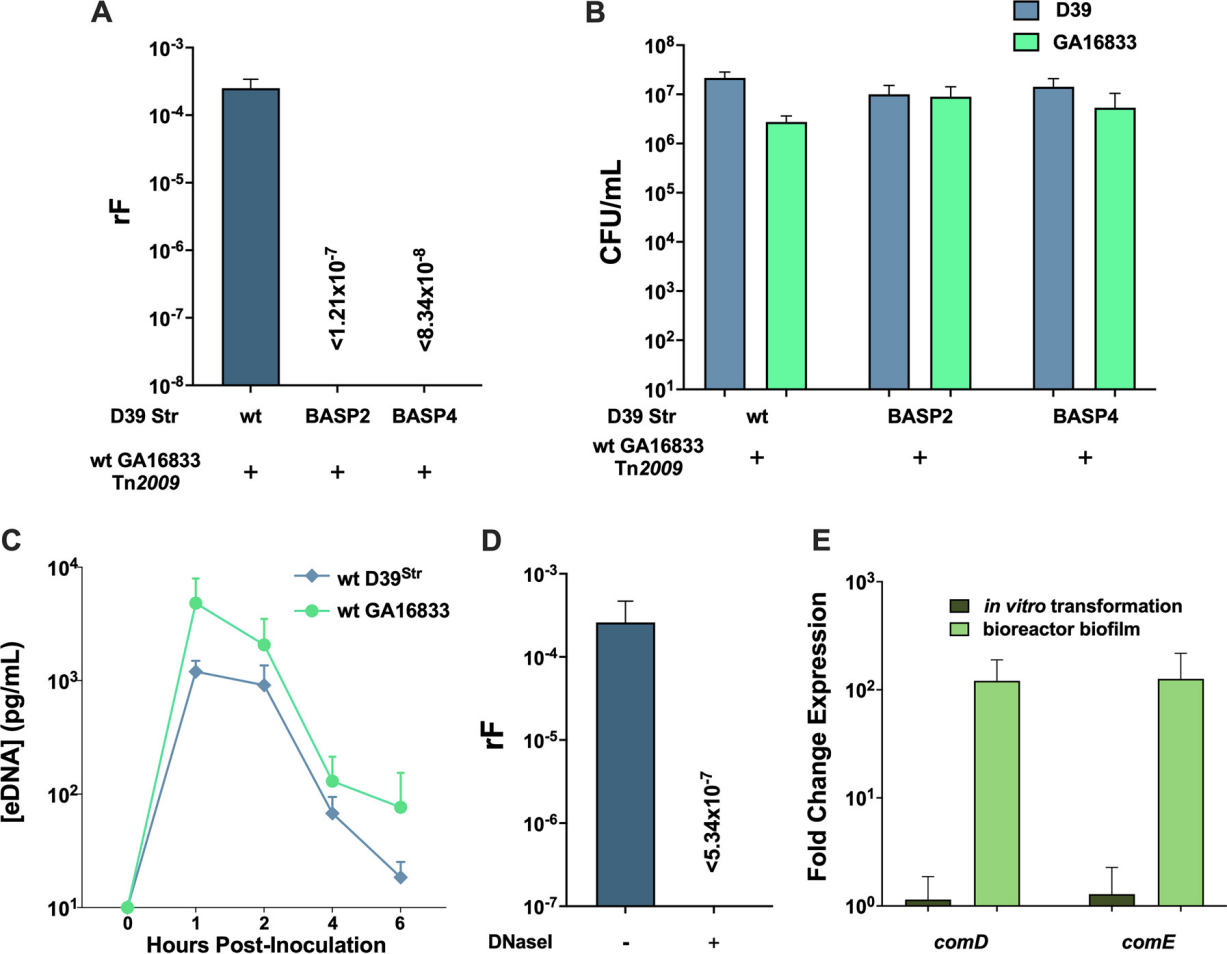
Pneumococcal Tn*2009* transfer in human nasopharyngeal biofilms requires competence and transformation machinery in the recipient. (A) Wild-type recipient D39^Str^, BASP2 (D39 Δ*comE*), or BASP4 (D39 Δ*comEA*/*EC*) were coinoculated with wild-type donor GA16833^Tet/Ery^ in the bioreactor. The data represent recombination frequencies for D39 uptake of Tet resistance. (B) Total density (CFU per milliliter) of each strain in the bioreactor coinoculations was calculated by dilution plating. (C) Extracellular DNA from spent medium of the D39^Str^ and GA16833^Tet/Ery^ bioreactor coinoculation was quantified by serotype-specific qPCR for serotypes 2 and 19F. (D) Wild-type D39^Str^ and GA16833^Tet/Ery^ bioreactor coinoculations were incubated in the presence or absence of 20 U/mL DNase I, and recombination frequencies were calculated for D39 uptake of Tet resistance. (E) Fold change in early competence gene expression of *comD* and *comE* in recipient strain D39^Ery/Str^ was calculated from bioreactor coinoculation of D39^Ery/Str^ and BASP1 normalized to an *in vitro* transformation without CSP addition.

Natural competence for DNA uptake via transformation is an important mechanism for horizontal gene transfer in *S. pneumoniae*. Early competence development depends on expression of the *comCDE* operon, encoding a competence-stimulating peptide (CSP), ComC, a histidine kinase, ComD, and a response regulator, ComE (19, 36). Recipient BASP2 (D39 *ΔcomE*), confirmed by *in vitro* transformation to be incompetent in acquiring point mutations, was examined in the bioreactor with donor GA16833. As shown in Fig. 4A, no Tet-resistant recombinants were observed (rF of <1.21 × 10^−7^ ± 6.35 × 10^−8^), suggesting that recipient competence initiation was critical for Tn*2009* uptake.

The involvement of two late competence proteins, ComEA and ComEC, was also examined. ComEA is a DNA receptor that binds double-stranded DNA captured by the type IV pilus as it retracts, and the ComEC protein channel subsequently imports single-stranded DNA fragments (37). The *comEA* and *comEC* genes, which are transcribed in tandem, were deleted and replaced with the *cat* cassette in strain D39^Str^. The recipient D39 *ΔcomEA*/*EC*::*cat*^Str^ double mutant (BASP4), when coinoculated with donor GA16833 in the bioreactor, yielded no detectable Tet-resistant recombinants and a rF of <8.34 × 10^−8^ ± 4.16 × 10^−8^ (Fig. 4A). When recovered from bioreactor experiments, BASP2 and BASP4 recipients were at similar cell counts to that of donor GA16833, indicating that rF reductions were not caused by growth defects or lower mutant densities relative to the donor strain (Fig. 4B).

As extracellular DNA (eDNA) levels influence transformation efficiency, we recovered eDNA in spent medium from the bioreactor experiments (20) and quantified eDNA concentrations of each strain using qPCR with serogroup-specific primers. Comparable eDNA concentrations were recovered from both wild-type donor GA16833 and wild-type recipient D39^Str^ (Fig. 4C) as well as for wild-type donor GA16833 and recipient BASP2 (Fig. S1). Additionally, the consequence of eDNA degradation was investigated by treating the biofilm with exogenous DNase I during a 6-h incubation. Compared to the parallel positive control of no treatment (rF of 2.60 × 10^−4^ ± 2.08 × 10^−4^), DNase I treatment resulted in no recombinant recovery, with an estimated rF of <5.34 × 10^−7^ ± 4.34 × 10^−7^ (Fig. 4D). Thus, eDNA degradation eliminated Tn*2009* transfer.

The increase in rF of Tn*2009* transfer in the bioreactor relative to that observed by *in vitro* transformation implied differential competence development under these two conditions, and we hypothesized that competence gene expression would be higher in the bioreactor environment. As no synthetic CSP was added in the bioreactor, the comparison was made with *in vitro* transformation reactions without CSP added. Coinoculating in the bioreactor a D39^Ery/Str^ recipient and the BASP1 donor with a *ΔcomCDE* deletion to avoid detecting donor *com* gene expression, *comD* and *comE* expression in the D39 recipient was about 120-fold greater in the bioreactor biofilm than under the *in vitro* transformation condition (Fig. 4E). Interestingly, this recipient upregulation of early *com* genes in the bioreactor biofilm was also much greater than that of classic *in vitro* transformation reactions, where 100 ng/mL synthetic CSP induced 83- and 57-fold increases in *comD* and *comE* expression, respectively, compared to reactions without CSP addition. The higher recipient competence gene expression in the donor GA16833 and recipient D39 mixed biofilm formed in the absence of exogenously added CSP supported a more robust competent state of D39 recipient cells relative to *in vitro* transformation conditions, which corroborated the efficient biofilm-mediated transfer of Tn*2009*.

### Integration of intact Tn*916*-related ICEs into the recipient pneumococcal genome occurred by homologous recombination.

The assumption that the Tet-resistant recombinants with the recipient genotype had integrated the entire Tn*2009* was confirmed by whole-genome sequencing (WGS). To analyze the extent of Tn*2009* integration in the recipient genome, four Tn*2009* recombinants independently recovered from selections on Tet+Str, Ery+Str, Tet+Ery+Str, and Ery+Str+Tmp were first characterized in detail. Quellung reactions and serotype-specific conventional PCRs confirmed that the recombinants were serotype 2 of the D39 recipient. Multilocus sequence typing (MLST) analysis of seven housekeeping genetic loci (38) showed all recombinants were sequence type 595 (ST595) of recipient D39 and not ST236 of donor GA16833 (Table 2), confirming that the recombinants were of the D39 genetic lineage and not a result of GA16833 undergoing capsule switching.

**TABLE 2:** Homologous recombination of donor DNA fragments of various sizes with intact Tn*2009* into D39 bioreactor recombinant genomes

Donor (ICE, MLST)	Recipient (MLST)	Recombinant (MLST)	Size of recombined donor fragment with ICE (bp)
GA16833 (Tn*2009*^Tet/Ery^, ST236)	D39^Str/Tmp^ (ST595)	Tet + Str (ST595)	33,176
GA16833 (Tn*2009*^Tet/Ery^, ST236)	D39^Str/Tmp^ (ST595)	Tet + Ery + Str (ST595)	35,566
GA16833 (Tn*2009*^Tet/Ery^, ST236)	D39^Str/Tmp^ (ST595)	Ery + Str (ST595)	55,152
GA16833 (Tn*2009*^Tet/Ery^, ST236)	D39^Str/Tmp^ (ST595)	Ery + Str + Tmp (ST595)	40,575

WGS of D39 recombinants was performed to probe the extent of recombination. WGS data confirmed that all four recombinants indeed harbored the entire 23.5-kb Tn*2009* integrated at the same genomic locus as the GA16833 donor. There was an ~9.5-kb genome sequence of D39 replaced by Tn*2009* in the GA16833 genome, and this expected deletion was also confirmed in the recombinants. Based on donor-specific SNP distributions, donor DNA fragments of various sizes flanking Tn*2009* were detected in the recombinant genomes. As shown in Table 2, there was an ~33.2-kb donor DNA fragment incorporated in the Tet+Str recombinant, while the Ery+Str recombinant had an insertion of an ~55.2-kb donor DNA fragment. The Tet+Ery+Str-selected recombinant carried Tn*2009* on an ~35.6-kb donor DNA fragment, whereas the recombinant from the Ery+Str+Tmp selection had incorporated an ~40.6-kb donor DNA fragment (Table 2). Thus, these Tn*2009*-containing D39 recombinants incorporated donor DNA fragments that ranged from ~33 kb to ~55 kb. Genomes of two Tn*2010*-containing D39 recombinants, independently selected on Tet+Str and Ery+Str after the coinoculation with donor GA47281, were also examined. Donor DNA fragments of ~38.4 and ~34.5 kb containing the intact Tn*2010* were identified, respectively (Table S4). Conjugative transfer of prototype Tn*916* is expected to result in site-specific recombination with a precise excision and integration of Tn*916* flanked by coupling sequences, thus incapable of transferring additional flanking SNPs from the donor genome (39). The variable donor DNA lengths indicated that integration of Tn*2009* and Tn*2010* into pneumococcal genomes occurred by homologous recombination.

**FIG 5:**
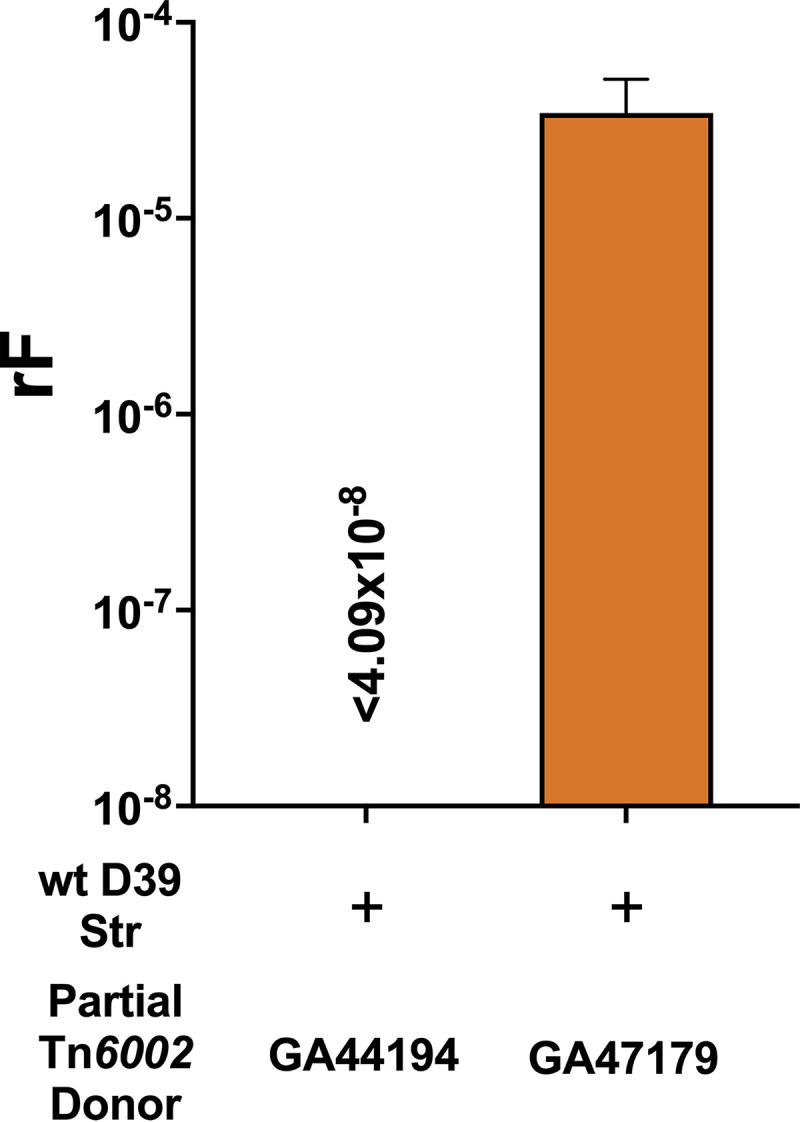
Partial pneumococcal Tn*6002* lacking conjugative regulatory mechanism transfers via transformation and homologous recombination in a strain-dependent manner. Wild-type recipient D39^Str^ and GA44194^Tet/Ery^ (with partial Tn*6002*, serotype 19A) or GA47179^Tet/Ery^ (with partial Tn*6002*, serotype 15A) were coinoculated in the bioreactor at 35°C. After a 6-h total incubation, recombination frequencies for D39 uptake of the tetracycline (Tet) resistance marker were calculated.

Additionally, we identified two clinical isolates, GA47179 (serotype 15A) and GA44194 (serotype 19A), each containing an incomplete copy of Tn*6002* (~17.0 kb), that retained both *ermB* and *tetM*. Tn*6002* in these isolates was truncated ~100 bp downstream of *tetM*, thus missing critical conjugative *xis* and *int* genes. Bioreactor experiments with donor GA44194 recovered only serotype 19A recombinants, indicating that GA44194 acquired Str resistance more efficiently from D39^Str^ leading to a rF of <4.09 × 10^−8^ ± 3.40 × 10^−8^ for uptake of Tet resistance by the designated D39 recipient (Fig. 5). Utilizing recipient D39^Ery/Str^ and donor GA44194 *ΔcomCDE*::*cat*^Str^ (BASP8), we detected that *comD* expression in the D39 recipient was about 10.8-fold greater, while *comE* expression showed no changes (0.7-fold) in the biofilm compared to the *in vitro* transformation condition without synthetic CSP (Table 3). Thus, when coinoculated with donor BASP8, the induction of *com* gene expression in the D39^Ery/Str^ recipient was significantly lower than that obtained with donor BASP1 (Fig. 4E). These data suggested a less competent D39 recipient with the GA44194 donor strain, potentially contributing to the lower rF. When examined with the bioreactor system, Tet resistance of another partial Tn*6002* donor, GA47179, was transferred to recipient D39^Str^ at a rF of 3.44 × 10^−5^ ± 1.69 × 10^−5^ (Fig. 5). WGS and SNP analyses of a Tet+Str-selected D39 recombinant found that Tn*6002* was incorporated within an estimated donor length of ~101.0 kb (Table S4). The strain-dependent, efficient transfer of an incomplete Tn*6002* lacking critical conjugation genes again supported the horizontal transfer of large ICE-containing DNA fragments between pneumococcal strains via transformation and homologous recombination.

**TABLE 3:** Early *com* gene expression in recipient D39^Ery/Str^ from dual-strain bioreactor biofilm with BASP8 compared to *in vitro* transformation without CSP addition

Strain	Expression (SD^*a*^) of:	
	*comD*	*comE*
D39^Ery/Str^	10.81 (14.83)	0.70 (0.14)

a SD represents the standard deviation from two independent biological replicates.

**FIG 6:**
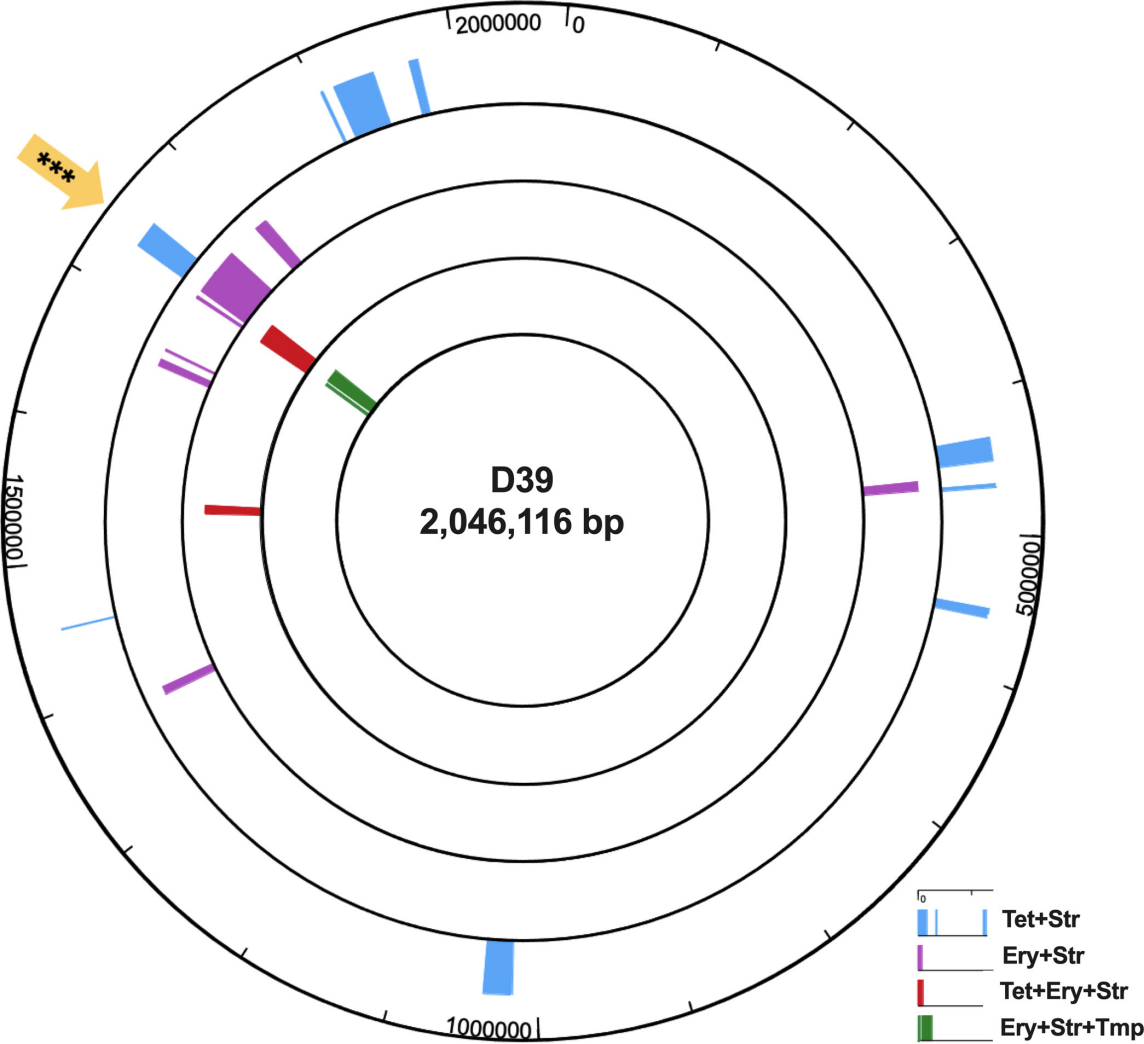
Whole-genome sequencing reveals homologous recombination of large Tn*2009*-containing fragments (~33 to ~55 kb) from GA16833 into D39 bioreactor recombinants. Bioreactor recombinants from the GA16833 and D39^Str/Tmp^ coinoculation were mapped against the recipient strain D39^Str/Tmp^. Donor GA16833 DNA recombination regions, denoted by the different colored blocks, were determined by locating donor SNP clusters flanked by recipient sequence using variant SNP analyses. A yellow block arrow with asterisks denotes the blocks representing the recombined donor fragments containing Tn*2009*.

Congression, the cotransformation of distinct unlinked fragments of DNA into the same cell, can occur during transformation. Although rare, congression has been previously demonstrated in *S. pneumoniae* (40). To detect additional independent recombination events distant from integration of the ICE-containing fragments, we conducted whole-genome variant analyses. SNPs transferred from the donor GA16833 genome into the recombinants were identified, and the presence of multiple consecutive GA16833 SNPs flanked by extensive recipient D39 sequence was considered incorporation of a donor DNA fragment and, thus, a possible transformation event. The outermost 5′ and 3′ donor SNPs were then used to estimate the minimal donor DNA length recombined into the recipient genome. Multiple possible cotransformation events at various genomic locations in each of the four Tn*2009* recombinant genomes were detected (Fig. 6).

### Efficient transfer of Tn*916*-related ICEs occurred between other *S. pneumoniae* donors and recipients in human nasopharyngeal biofilms.

**FIG 7:**
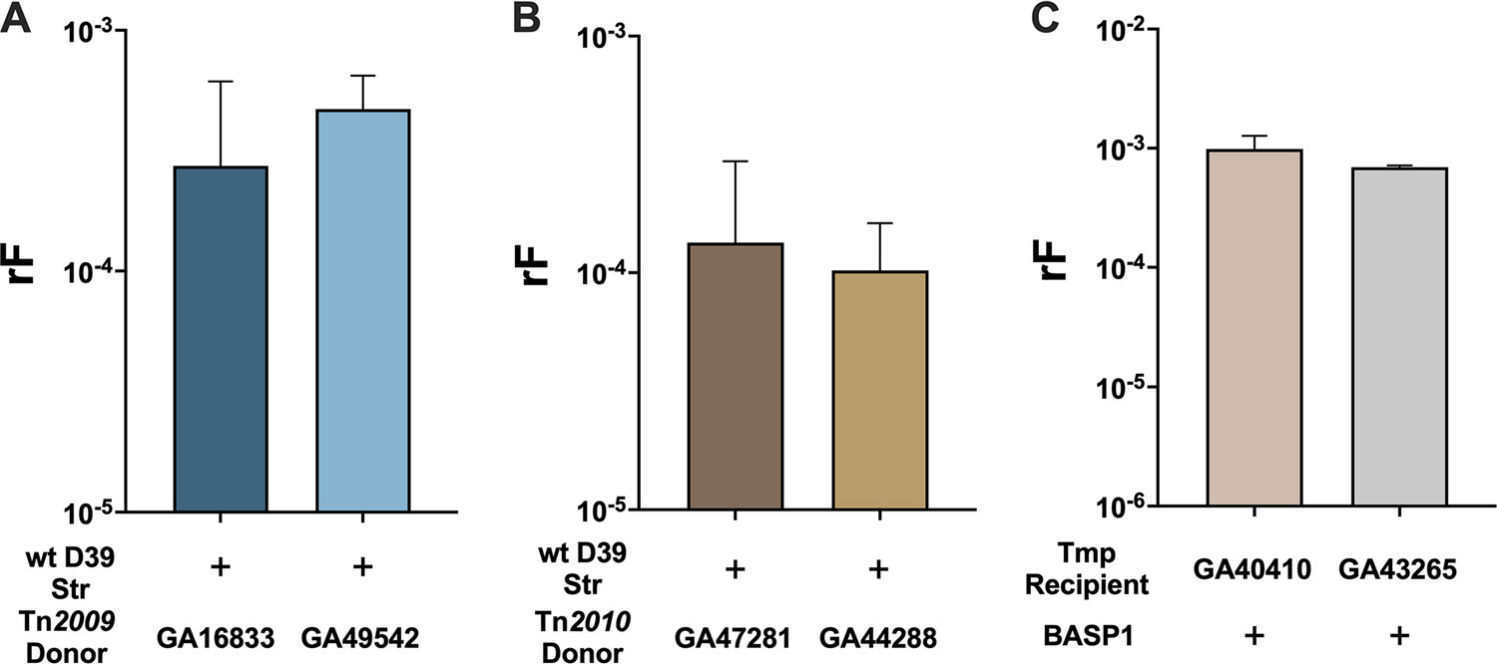
Efficient transfer of Tn*916*-related ICEs via transformation and homologous recombination in nasopharyngeal biofilms occurs in other *S. pneumoniae* strains. Pneumococcal recipients and Tn*916*-related ICE-containing donor isolates were coinoculated at 35°C in the bioreactor. After a 6-h total incubation, recombination frequencies were then calculated for recipient uptake of Tet resistance conferred by Tn*916*-related ICEs via selection of recombinants on Tet+Str or Tet+Tmp. (A) Wild-type D39^Str^ and wild-type GA16833^Tet/Ery^ or GA49542^Tet/Ery^ (Tn*2009*); (B) wild-type D39^Str^ and wild-type GA47281^Tet/Ery^ or GA44288^Tet/Ery^ (Tn*2010*); (C) wild-type GA40410^Tmp^ or GA43265^Tmp^ and BASP1 (Tn*2009*).

To assess whether Tn*916*-related ICE dissemination via transformation was widely applicable in *S. pneumoniae*, additional Tn*2009*- and Tn*2010*-containing *S. pneumoniae* clinical isolates were studied in the bioreactor using the Tet+Str selection. Tn*2009* was integrated in SP_1638 (TIGR4 annotation) in GA49542 (serotype 9V), distinct from GA16833 with Tn*2009* integrated in SP_1947 (TIGR4 annotation). When donor GA49542 was coinoculated with recipient D39^Str^ in the bioreactor, we observed a rF of 4.71 × 10^−4^ ± 1.77 × 10^−4^ (Fig. 7A) for Tn*2009* transfer, similar to the rF obtained with donor GA16833. We also investigated another strain, GA44288 (serotype 19A), with Tn*2010* incorporated into the same genomic locus as in GA47281 (serotype 19F). The rF for recipient D39^Str^ uptake of Tn*2010* from donor GA44288 was 1.02 × 10^−4^ ± 5.96 × 10^−5^, comparable to that obtained from donor GA47281 (Fig. 7B). WGS analysis of three independent Tet+Str recombinants indicated that the donor fragments encompassing Tn*2010* were 43.4, 35.2, and 44.5 kb in size (Table S4). Thus, Tn*2009* and Tn*2010* were transferred from multiple donor strains to recipient D39 efficiently (rF of ~10^−4^), indicating that the dissemination of Tn*916*-related pneumococcal ICEs in biofilms was independent of donor genomic lineages and the genomic location of ICEs.

We also sought to determine if the two serotype 19A Tmp-resistant clinical isolates, GA40410 and GA43265, examined as recipients in mating experiments, could acquire ICEs as efficiently as D39. To prevent trimethoprim resistance marker uptake by the donor, we used the competence-deficient donor BASP1. Tn*2009* was efficiently taken up by recipient GA40410 at a recombination frequency of 9.87 × 10^−4^ ± 2.80 × 10^−4^, while recipient GA43265 incorporated Tn*2009* at a rF of 6.92 × 10^−4^ ± 2.41 × 10^−5^ (Fig. 7C). WGS analysis indicated that GA40410 recombinants incorporated Tn*2009* on donor fragments of 40.5, 73.7, and 92.3 kb, while GA43265 recombinants incorporated Tn*2009* on fragments of 89.6 and 50.6 kb in size (Table S4). Together, these data demonstrated efficient Tn*916*-related ICE transfer between multiple *S. pneumoniae* strains in a dual-strain, nasopharyngeal biofilm via transformation and homologous recombination.

## DISCUSSION

The continued emergence of multidrug resistance in *S. pneumoniae* limits treatment options for invasive pneumococcal disease. Thus, a WHO initiative, launched in 2017, emphasizes research into pneumococcal antibiotic resistance mechanisms and development of new antipneumococcal agents (6). The mechanism of high-frequency horizontal transfer of >20-kb ICEs of the Tn*916* family, such as Tn*2009*, Tn*6002*, and Tn*2010*, which carry the important resistance genes *tetM*, *mefE*/*mel*, and/or *ermB* in *S. pneumoniae*, was addressed in the present study.

During colonization, which can last for months, the pneumococcus forms highly organized biofilms on the epithelial surface of the human nasopharynx (41). Approximately 50% of children carrying *S. pneumoniae* can be colonized by two different pneumococcal serotypes, and up to five cocolonizing pneumococcal serotypes can be detected at any one time (42–44). We found that >20-kb Tn*916*-related ICEs horizontally disseminate at high frequencies between *S. pneumoniae* in dual-strain biofilms (rF of 10^−4^) established on a human nasopharyngeal cell monolayer and in a continuous flow bioreactor system, which mimics the microenvironment of the human respiratory epithelium. Previous work using the bioreactor has shown high rFs for the exchange of smaller antibiotic resistance determinants between *S. pneumoniae* strains (20, 45).

Conjugation is a transfer mechanism for ICEs in several bacterial species, including *S. pneumoniae*. Examples of interspecies conjugation include those of pneumococcal ICEs Tn*6002* (20.8 kb) into Streptococcus pyogenes (cF of ~10^−8^) (10), Tn*6003* (25.1 kb) into Enterococcus faecalis (cF of ~10^−7^) (10), and Tn*1207.3* (52.5 kb) into S. pyogenes (cF of ~10^−3^) or Streptococcus gordonii (cF ~10^−4^) (46). Additionally, the large composite Tn*5253* (64.5 kb) conjugates to pneumococcal recipients at cFs ranging from ~10^−7^ to ~10^−4^ (47), and Tn*5251*, usually found within Tn*5253*, has been shown to conjugate independently from a pneumococcal Tn*5253* donor to *S. pneumoniae* TIGR4 (cF of ~10^−5^) (15). While we demonstrated Tn*916* conjugation between B. subtilis strains at frequencies of 10^−5^, conjugative transfer of a pneumococcal Tn*916*-related ICE, Tn*2009*, was not detected between *S. pneumoniae* under similar mating conditions (cF of <10^−8^). Tetracycline induces expression of conjugative genes on Tn*916*, resulting in formation of circular intermediates and subsequent transfer of Tn*916* to recipient cells (24, 25). However, conjugative gene expression in Tn*2009* was not induced by tetracycline, likely due to insertion of the Mega element downstream of *tetM*, disrupting the regulatory coupling between *tetM* and conjugative genes. In addition, we did not detect Tn*2009* CI formation and Tn*2009* transfer in nasopharyngeal biofilms was not affected by a deletion of the critical conjugative gene *ftsK*. Collectively, these data demonstrated that conjugation is not the underlying mechanism for the high-frequency transfer of pneumococcal ICEs in biofilms on human nasopharyngeal cells.

Another potential horizontal gene transfer method is transduction mediated by bacteriophages. Prophages are abundantly present in pneumococcal genomes and contribute to virulence and pneumococcal physiology (48, 49). However, the importance of phage transduction in the spread of antibiotic resistance in *S. pneumoniae* is much less clear. Wyres et al. identified a pneumococcal 1968 isolate, 18C/3, carrying a Tn*916*-related ICE that is associated with a streptococcal phage similar to Streptococcus phage 040922 (50). We found no evidence that Tn*2009*, Tn*6002*, and Tn*2010* carried by the donor strains examined in this study were part of or were associated with phage elements. Furthermore, the variable sizes of donor DNA fragments containing ICEs identified in the recombinant genomes did not support phage-mediated transfer of Tn*916*-related ICEs.

Transformation is the major mechanism of horizontal genetic exchange in naturally competent *S. pneumoniae*. *In vitro* transformation experiments using mixtures of planktonic pneumococci, exogenous synthetic CSP, and purified DNA generally yield frequencies of 10^−4^ to 10^−6^ with the uptake of ~2- to ~6-kb DNA fragments (51–54). Our data reproduced these observations, in which recipients D39 and TIGR4 were transformed with point mutation-mediated Tmp or Str resistance at frequencies of 10^−5^ per μg of DNA. However, analogous experiments using purified ICE-containing genomic DNA did not yield Tet-resistant transformants (rF < 10^−7^), and this was not caused by a DNA fragment size limitation.

Previously, Cowley et al. noted that an environment of close cell-to-cell contact, which consisted of pneumococcal coincubation on filters or mixed cultures forming mature biofilms, led to transformation of 8- to 30-kb DNA fragments (40). Additionally, serotype switching events were a result of transformation of 22- to 39-kb DNA fragments (55). We found that within biofilms on human nasopharyngeal cells, Tn*916*-related ICEs transferred efficiently to pneumococcal recipients, resulting in acquisition of these large antibiotic resistance elements at a rF of ~10^−4^. Similar results were shown with multiple *S. pneumoniae* donors, including two donors of Tn*2009*, two of Tn*2010*, and one of Tn*6002*, as well as with four recipient strains, D39^Str/Tmp^, D39^Str^, GA40410^Tmp^, and GA43265^Tmp^.

Efficient transfer of Tn*916*-related ICEs likely requires close contact between donor and recipient *S. pneumoniae* strains in mixed biofilms (40), an environment likely found during nasopharyngeal colonization (56). The temperature maintained for the bioreactor (35°C) is lower than the *in vitro* transformation experiments (37°C). *In vitro* transformation conducted at 35°C resulted in a lower rF for streptomycin resistance uptake than that at 37°C, while no uptake of Tn*2009*-encoded tetracycline resistance was detected at either temperature (see Fig. S3 in the supplemental material). Thus, the difference in temperatures is unlikely to account for the efficient ICE transfer within dual-strain nasopharyngeal biofilms. Preliminary data from single-strain recipient D39^Tmp^ or D39^Str^ biofilms formed on nasopharyngeal cells and exposed to extracellular genomic DNA supplied in the flow medium resulted in the uptake of streptomycin resistance at a rF of 1.66 × 10^−6^ per μg DNA, but not of Tn*2009*-mediated tetracycline resistance (rF of <7.68 × 10^−8^ per μg DNA) (Fig. S2). These data further support that close interaction between different donor and recipient strains within the dual-strain biofilms is needed for efficient transformation of Tn*916*-related ICEs. Induction of the competent state in *S. pneumoniae* initiates DNA release from a subfraction of different *S. pneumoniae* populations, potentially via cell lysis (57), and fratricide is critical for efficient gene transfer between donor and recipient pneumococci in biofilms (58). Overall, this close contact, donor-recipient interactive environment is absent in the classic transformation of planktonic cells.

Whole-genome sequencing and minimum recombination junctions defined by SNP analysis confirmed the integration of very large donor DNA fragments containing intact ICE elements. For Tn*2009*, donor fragments were estimated to range from ~33 to ~55 kb in size, while Tn*2010* was transferred on ~34- to ~45-kb DNA from two donor strains. Finally, we observed that the partial Tn*6002* ICE missing critical conjugation genes recombined on a donor fragment size of ~101 kb. Evidence of multiple homologous recombination events, or congression, was also detected in several Tn*2009*-containing recombinants.

*S. pneumoniae* develops a naturally competent state through a positive feedback mechanism involving the ComCDE signaling cascade (19, 36). Upon pneumococcal colonization of the upper respiratory tract, biofilms are formed with upregulation of competence genes (59–61). We detected 120-fold increases of *comD* and *comE* expression in the D39 recipient recovered from the mixed nasopharyngeal biofilm compared to planktonic D39 cultures. Deletions of *comE* or *comEA* and *comEC* in the D39 recipient eliminated recovery of Tn*2009*-containing D39 recombinants. The elimination of recombinants after DNase I treatment further supported transformation as the mechanism responsible for efficient ICE transfer in the biofilms.

In conclusion, efficient Tn*916*-related ICE dissemination in *S. pneumoniae* was demonstrated in human nasopharyngeal cell biofilms via transformation and homologous recombination of large DNA fragments. High-frequency transfer of Tn*916*-related ICEs in biofilms occurred in multiple combinations of pneumococcal donors and recipients. A biofilm environment with close contact of pneumococcal cells and the consequent upregulation of the competence pathway were critical for supporting horizontal dissemination of >20-kb Tn*916*-related ICEs and the antibiotic resistance determinants of these elements. Efficient transformation in mixed biofilms that mimic a natural nasopharyngeal colonization environment supports the epidemiological observations of widespread dissemination of *S. pneumoniae* ICEs in the pneumococcal population.

## MATERIALS AND METHODS

### Bacterial strains, culture media, and antibiotics.

The strains of *S. pneumoniae* used in this study are listed in Table S1 in the supplemental material. Clinical isolates were provided by the Georgia Emerging Infections Program. All *S. pneumoniae* strains were cultured on blood agar plates or with Todd-Hewitt broth with yeast extract (THY broth) and grown at 37°C with 5% CO_2_. B. subtilis strains were cultured on LB plates or LB broth at 37°C. Where indicated, the following antibiotics were utilized for preparation of antibiotic agar plates: tetracycline (1 or 2 μg/mL), streptomycin (100, 200, and 220 μg/mL), trimethoprim (14 μg/mL), chloramphenicol (4.5 μg/mL), erythromycin (0.5 μg/mL), and spectinomycin (100 μg/mL). All antibiotics were purchased from Millipore-Sigma (Saint Louis, MO).

### Mutant construction.

Mutation constructs were created by sequential overlapping PCR or splicing by overlap extension (SOE) PCR. GA16833^Tet/Ery^ served as the parental strain for the conjugation mutant (*ΔftsK*), while D39^Str^ and D39^Str/Tmp^ were the parental strains for competence (*ΔcomE* or *ΔcomCDE*) and transformation (*ΔcomEA*/*EC*) mutants. The primers utilized are listed in Table S2. The chloramphenicol resistance gene, *cat*, was amplified from pEVP3 (62). Purified genomic DNA was utilized as the template to amplify the upstream (5′-end) and downstream (3′-end) regions of the target gene using the corresponding primers carrying the necessary overlapping sequences. These individual DNA fragments were amplified using Q5 high-fidelity polymerase (New England Biolabs, Ipswich, MA). Mixtures of two fragments (5′ end plus *cat* or *cat* plus 3′ end) were used as the template for the first round of overlapping PCRs. The third fragment was then linked by a second PCR using either One *Taq* (New England Biolabs) or *Taq* (Roche Diagnostics, Indianapolis, IN) DNA polymerase plus *Taq* Extender PCR additive (Agilent, Santa Clara, CA) to generate the final construct. PCR products purified with the Zymo DNA Clean and Concentrator kit (Irvine, CA) were sequenced to confirm the desired mutation. Purified PCR constructs (0.5 to 1 μg) were used to transform precompetent cells by *in vitro* transformation. Transformants selected on 4.5 μg/mL chloramphenicol were sequenced to confirm the presence of the intended mutation.

To generate the *ΔftsK* mutant, an upstream region was amplified with BSA17 and BSA18 and a downstream region was amplified with BSA19.1 and BSA20. The chloramphenicol resistance marker *cat* was amplified with primers EVP3_CmF and EVP3_CmR. SOE PCR was performed using three individual PCR products and with the outermost primers BSA17.1 and BSA20.1 to create the Δ*ftsK*::*cat* cassette. The *ΔcomE* mutant was generated by amplifying a *comE* upstream region with MS93 and MS99, a *comE* downstream region with MS100 and MS96, and the *cat* gene with MS101 and MS102. The final Δ*comE*::*cat* cassette was obtained by SOE PCR with primers SL107 and SL108. The *ΔcomEA*/*EC* mutant was created by amplifying a *comEA* upstream region with BSA1a and comEA_5RA3, a *comEC* downstream region with comEC_3FA3 and BSA6a, and the *cat* gene with EVP3_CmF and EVP3_CmR. Primers BSA11a and BSA6a were utilized in SOE PCR to obtain the final Δ*comEA*/*EC*::*cat* cassette. Finally, the Δ*comCDE* mutant was created by amplifying an upstream region of *comC* with MS96 and SL115, a downstream region of *comE* with SL118 and SL119, and the *cat* gene with SL116 and SL117. Primers MS96 and SL119 were utilized to generate the final Δ*comCDE*::*cat* construct by SOE PCR.

### *In vitro* transformation assay.

D39 and TIGR4 pneumococcal strains were made precompetent using standard methods (63). Briefly, an overnight plate culture was used to inoculate complete transformation medium (CTM) and grown to an optical density at 600 nm (OD_600_) of 0.6 to 0.7. This primary culture was used to make a 1:20-diluted secondary culture, which was grown to OD_600_ of 0.35 to 0.45 (mid-log phase). Glycerol was then added to the competent cell aliquots at a final concentration of 10% (vol/vol) and stored at −80°C. These precompetent pneumococcal cells were transformed in CTM using 500 ng of purified genomic DNA and 100 ng/mL of synthetic CSP1 or CSP2 in 200- or 300-μL total reaction volumes. The CSPs were synthesized by Millipore-Sigma (Saint Louis, MO), the Emory University Microchemical Facility (64), or GenScript (Piscataway, NJ). Recombination frequencies for *in vitro* transformation reactions were calculated as number of transformants on antibiotic selection plate divided by the total population of *S. pneumoniae* cells recovered on nonselective blood agar plate and normalized to micrograms of transforming DNA.

### Cell culture.

Human pharyngeal cells (Detroit 562; ATCC CCL-138) were maintained using 1× minimum essential medium (MEM) supplemented with 10% fetal bovine serum (FBS), 1% nonessential amino acids (100×), 1% l-glutamine (200 mM), 1% HEPES buffer (1 M), and 1% penicillin-streptomycin (10,000 U/mL). All cell culture media and supplements were from Life Technologies, Gibco (Gaithersburg, MD). For routine passaging of cells, 0.25% trypsin-EDTA (1 mM) was used to lift the cells. Cells were incubated in a sterile incubator at 37°C with 5% CO_2_.

### Human nasopharyngeal biofilm bioreactor coinoculations.

A confluent monolayer of Detroit 562 cells (ATCC CCL-138) was grown on a Corning Snapwell with a 0.4-μm-pore polyester membrane (Corning, NY). These Snapwells were placed inside a sterile vertical diffusion chamber from the bioreactor, allowing the Detroit 562 cells to rest on the apical side (inner chamber) and to be perfused with flowing bioreactor medium composed of 1× MEM supplemented with 5% FBS, 1% nonessential amino acids (100×), 1% l-glutamine (200 mM), and 1% HEPES buffer (1 M). The flow of bioreactor medium at a rate of ~0.2 mL/min was generated by a Cole Parmer Master Flex L/S peristaltic pump (Vernon Hills, IL). Where indicated, 20 U/mL of DNase I was added to the bioreactor medium. Spent medium (collected for eDNA quantification as described below) and planktonic cells exited the bioreactor chamber through a parallel outlet located at the top of the chamber (20).

For the coinoculation, overnight plate cultures of each pneumococcal strain were washed three times with 1× Dulbecco’s phosphate-buffered saline (DPBS) and resuspended in 500 μL of 1× DPBS, and the OD_600_ was measured. Appropriate volumes of bacteria were mixed to make a suspension of equal densities at OD_600_ of 0.1 (10^6^ CFU/mL each) and inoculated through the apical perfusion path of the bioreactor chamber. After a static 1-h incubation at 35°C to allow for *S. pneumoniae* adherence to Detroit 562 cells, the flow of medium was initiated and continued for another 5 h. At the conclusion of the incubation period, the Snapwells were removed and the dual-strain biofilms formed on the Detroit 562 cells were gently washed and sonicated for 20 s in a Branson ultrasonic water bath (Danbury, CT). The bacteria were suspended by extensive pipetting and vortexing, and serial dilutions in 1× DPBS were performed. To obtain the total population for each strain in the coinoculations, serial dilutions were plated on antibiotic plates unique to each individual strain. The cells of the total recombinant population were plated on dual- or triple-antibiotic selection plates. The recombination frequencies (rFs) for bioreactor experiments were calculated as follows: (total number of recombinants × serotype proportion of recombinants)/total population for specific serotype (20).

### DNA extraction and serotype-specific qPCR.

Recombinants recovered from bioreactor experiments were pooled in 200 μL of sterile 1× DPBS. A lysis buffer containing 100 μL of TE buffer (10 mM Tris, 1 mM EDTA [pH 8.0]), 40 mg/mL of lysozyme, and 75 U/mL of mutanolysin was added to each of the samples and incubated at 37°C for 1 h. Two hundred microliters of Qiagen buffer AL and 20 μL of Qiagen proteinase K were added, followed by incubation at 56°C for 30 min. DNA was extracted using the Qiagen QIAamp DNA Mini Kit (Valencia, CA) per the manufacturer’s instructions. DNA was eluted in 100 μL of elution buffer and used for serotype-specific qPCR with the primers and probes listed in Table S2 as well as the Bio-Rad SsoAdvanced Universal Probes supermix (Hercules, CA). These reactions targeted the capsule (*cps*) locus of specific pneumococcal serotypes. The cycling conditions were as follows: 1 cycle at 95°C for 2 min, followed by 39 cycles of 95°C for 15 s and 60°C for 30 s. A standard curve for final genome equivalents per milliliter of each serotype was performed alongside the samples and consisted of serially diluted DNA standards corresponding to the following genome equivalents: 8.58 × 10^6^, 4.29 × 10^6^, 2.14 × 10^6^, 4.29 × 10^5^, 4.29 × 10^4^, 4.29 × 10^3^, 4.29 × 10^2^, 4.29 × 10^1^, 2.14 × 10^1^, and 2.14 (42, 65). The proportion of recombinants that belong to each serotype was calculated by dividing the number of genome equivalents for a specific serotype by the total sum of genome equivalents for both strains in the bioreactor coinoculations, and this value was utilized to calculate recombination frequencies (rFs).

### Alignments of pneumococcal Tn*916*-related ICEs with prototype Tn*916*.

BLASTN and -outfmt 6 were utilized for generating comparison files between ICEs. Along with GenBank (.gbk) files, the comparison files were inputted into EasyFig 2.2.2 (66) to produce sequence alignments as well as to determine percentages of identity.

### Gene expression studies with qRT-PCR.

For *tetM* and conjugative gene expression studies, single-strain broth cultures of *S. pneumoniae* strains and B. subtilis CMJ253 were grown in a primary culture to an OD_600_ of 0.50 to 0.65. The primary cultures were then diluted 1:20 to inoculate a secondary culture. The secondary cultures were incubated in the presence or absence of 2.5 μg/mL tetracycline for 2.5 h at 37°C. One milliliter of cultures (~10^8^ CFU) was pelleted at 16,400 rcf for 8 min at room temperature. Five hundred microliters of supernatant was discarded, and the remaining 500 μL supernatant was used to resuspend the bacterial pellets. One milliliter of Qiagen’s RNAprotect reagent was added, and the mixture was incubated at room temperature for 5 min. For single-donor strain or mixed donor-recipient broth cultures, *S. pneumoniae* strains were first grown separately in broth to OD_600_ of about 0.4. A mixture of 10^8^ CFU of both strains or a single-strain culture was incubated for another 2.5 h and then treated with 2 vol of RNAprotect reagent. For bioreactor experiments that underwent a 1-h static incubation and 5-h continuous flow incubation at 35°C, total biofilm bacteria suspended in 1× DPBS were collected at the end of the 6-h incubation period and treated with 2 vol of RNAprotect reagent, while *in vitro* transformation reactions with or without synthetic CSP were incubated at 37°C for 2 h and then treated with 2 vol of RNAprotect reagent. Following incubation, the samples were centrifuged at 12,000 rcf for 10 min, the supernatant was discarded, and the pellets were stored at −80°C.

Qiagen RNeasy Mini Kit’s protocol was followed, with the exception that 40 mg/mL of lysozyme and 75 U/mL of mutanolysin were used in TE buffer for bacterial cell lysis. RNA samples were treated with the TURBO DNA-free kit (Gaithersburg, MD) and subsequently purified and concentrated with the Zymo RNA Clean and Concentrator kit (Irvine, CA). All RNA samples were verified to be free of DNA contamination via conventional PCR using primers targeting *S. pneumoniae recA* or B. subtilis *orf20* (relaxase) (BSA25 and BSA26). cDNA was prepared using the Bio-Rad iScript reverse transcription supermix (Hercules, CA) and 350 ng, 500 ng, 800 ng, or 1 μg of purified RNA. Quantitative reverse transcription-PCR (RT-PCR) was conducted with the Bio-Rad iQ SYBR green supermix (Hercules, CA). All primers for the target and internal control genes were validated by the threshold cycle (2^−ΔΔ^*^Ct^*) method (67). The following cycle conditions were utilized on a Bio-Rad CFX96 Touch real-time PCR machine (Hercules, CA): 1 cycle at 95°C for 3 min, 40 cycles of 95°C for 15 s, 57°C or 60°C for 15 s, and 72°C for 30 s.

Fold change in expression was calculated using the 2^−ΔΔ^*^Ct^* method (67). For the tetracycline-induced and uninduced broth cultures of *S. pneumoniae* strains and B. subtilis CMJ253, 16S rRNA was utilized as the internal control and fold change was normalized to the uninduced condition. Due to 16S rRNA being present in both wild-type donor and recipient *S. pneumoniae* strains in the bioreactor, the internal control for conjugative gene expression was the donor-specific *cat* gene from BASP1. When analyzing expression of *com* genes specifically from the recipient strain, expression of *ermB* inserted in the nonessential *bgaA* locus of the D39^Ery/Str^ recipient served as the internal control for expression analysis of bioreactor biofilms.

### Quantification of ICE circular intermediates.

The formation of circular intermediates was examined under the following conditions: (i) broth cultures treated with or without 2.5 μg/mL tetracycline for 2.5 h at 37°C, (ii) 4-h mating reaction mixtures consisting of GA16833:BASP2 or CMJ253:CAL419 on a blood agar plate, and (iii) the bioreactor experiment including D39^Str^ and GA16833. One milliliter of each broth culture was pelleted down at 16,400 rcf for 10 min at room temperature, and the supernatants were discarded. The bacterial pellets were then resuspended in 200 μL of 1× DPBS for DNA extraction. For the mating experiment, bacteria on the blood agar plate were collected in 400 μL of 1× DPBS and aliquots of 200 μL were utilized for DNA extraction. Finally, dual-strain biofilms were collected from the bioreactor and underwent DNA extraction. DNA was extracted with the Qiagen QIAamp DNA Mini Kit (Valencia, CA).

qPCR of the circular junctions was performed with the Bio-Rad iQ SYBR green supermix (Hercules, CA). A standard curve using genomic DNA from B. subtilis strain LDW737 (27), containing a cloned copy of the circular junction sequence that is shared between the Tn*916* prototype and the pneumococcal Tn*916*-related ICEs, was performed with the following genome equivalents: 8.58 × 10^6^, 8.58 × 10^5^, 8.58 × 10^4^, 8.58 × 10^3^, 8.58 × 10^2^, 8.58 × 10^1^, 4.29 × 10^1^, and 4.29. For the broth samples, 1 μL of 30 ng/μL DNA was added to the reaction mixtures. For the mating and bioreactor biofilm samples, 1 μL of 20 ng/μL DNA was utilized as the template for the qPCRs with the following cycling conditions: 1 cycle at 95°C for 3 min, followed by 40 cycles of 95°C for 15 s, 53°C for 15 s, and 72°C for 30 s. The genome copy numbers were based on *ftsK* (1 chromosomal copy) quantification, and the data were calculated as copy number of circular junction per genome, or CI/chromosome.

### Mating experiments.

Strains were inoculated in LB broth or THY broth from overnight plate cultures and grown to the late-log phase. Donor and recipient strains were mixed at a 1:10 ratio of 10^8^/10^9^ CFU and centrifuged for 15 min at 3,000 rcf to pellet bacteria. The pellet was resuspended in 100 μL of LB or THY broth, and DNase I was added to the mating mixture at 10 μg/mL. The mixture was plated on blood agar plates and incubated at 37°C for 4 h. The mating mixture was collected and resuspended in 1 mL LB or THY broth with a 10% glycerol final concentration. Serial dilutions were performed, and multilayer plating was carried out as described previously (32). *S. pneumoniae* was selected with 2 μg/mL Tet and/or 220 μg/mL Str or 14 μg/mL Tmp, while B. subtilis was selected with 10 μg/mL Tet and/or 100 μg/mL spectinomycin or 100 μg/mL Str.

### Quantification of extracellular DNA.

Spent medium was collected from the outlets of the bioreactor chambers at h 1, 2, 4, and 6 of incubation for 1 h. The samples were centrifuged for 10 min at 15,000 rpm at 4°C (Eppendorf, Hauppauge, NY) and subsequently sterilized with a 0.45-μm-pore filter. Extracellular DNA was extracted from 400 μL of the sterile supernatant samples using the Qiagen QIAamp DNA Mini Kit following the instructions starting from addition of ethanol to the samples. The eDNA was eluted in 100 μL of elution buffer and stored at −80°C for further use. The purified eDNA was utilized as the templates for serotype-specific qPCR as described above, and the standard curve was built using 1 × 10^3^, 1 × 10^2^, 1 × 10^1^, 1 × 10°, 1 × 10^−1^, 5 × 10^−2^, and 5 × 10^−3^ pg of chromosomal DNA corresponding to the appropriate serotype (20). The standard curve was used to calculate the eDNA concentrations for each time point sample utilizing the Bio-Rad CFX Manager software.

### Whole-genome sequencing and variant analysis.

Genomic DNA from bioreactor recombinants was purified using the Qiagen QIAamp DNA Mini Kit as instructed. Libraries were prepared utilizing the Illumina Nextera XT DNA library preparation kit (San Diego, CA) and sequenced by SeqCenter (Pittsburgh, PA) using the NextSeq 2000 platform. The paired-end read data were assembled and annotated using tools available on PATRIC's Bacterial and Viral Bioinformatics Resource Center. For variant analysis, the Illumina reads of the D39^Str/Tmp^ or D39^Str^ recipient strains were first mapped onto the closed genome sequence of reference strain D39 (NC_008533.2) using the DNAStar NGen program, and single nucleotide polymorphisms already present in the D39^Str/Tmp^ or D39^Str^ recipients were identified relative to the reference D39. These SNPs of D39^Str/Tmp^ or D39^Str^ were then discounted from the reference-guided assemblies of the recombinants to identify the remaining SNPs introduced by the donor strain. For the GA40410 and GA43265 recipient strains, the recombinants were assembled using individual WGS data as references to identify donor SNPs. Recombination blocks were estimated as clustering of consecutive donor SNPs flanked by recipient sequence. The outermost 5′ and 3′ donor SNPs were used to calculate the minimal length of the recombined donor DNA fragment.

### Statistical analysis.

All frequency, ratio, bacterial density, and eDNA concentration data were analyzed using two-tailed unpaired *t* tests on GraphPad Prism8.

### Data availability.

All data supporting the research findings of this study are included within the article and in the supplemental material. Annotated whole-genome sequences have been deposited in NCBI GenBank under BioProject no. PRJNA933161.
