# Supplementary material for: Dissemination of Tn916-Related Integrative and Conjugative Elements in Streptococcus pneumoniae Occurs by Transformation and Homologous Recombination in Nasopharyngeal Biofilms
Source: Microbiol Spectr. 2023 Mar 13;11(2):e03759-22. doi: 10.1128/spectrum.03759-22 (PMC10101023; doi:10.1128/spectrum.03759-22)
Supplement: Supplemental file 1 — Supplemental material. Download spectrum.03759-22-s0001.pdf, PDF file, 0.3 MB [file spectrum.03759-22-s0001.pdf]

Table S1. Strains used in this study.

Bacterial Strain	Description, Relevant Genotype and Phenotype	Reference or Source
Streptococcus pneumoniae		
WT D39	Avery strain, serotype 2	(1)
WT TIGR4	Invasive isolate, serotype 4	(2)
WT D39^{Str}	D39 derivative transformed with genomic DNA containing K56T point mutation in *rpsL*, Str resistant	This study
GA16833	Clinical isolate from Georgia Emerging Infections Program, serotype 19F, ICE Tn*2009* conferring Tet resistance (*tetM*), Ery resistance (*mefE*/*mel* on Mega)	(3)
GA47281	Clinical isolate from Georgia Emerging Infections Program, serotype 19F, ICE Tn*2010* conferring Tet resistance (*tetM*), Ery resistance (*ermB* and *mefE*/*mel* on Mega)	(3)
WT D39$^{Str/Tmp}$	D39 derivative transformed with genomic DNA containing K56T point mutation in *rpsL* (Str resistant) and I100L point mutation in *folA* (Tmp resistant)	(4)
BASP1	GA16833Δ*comCDE* – GA16833 derivative with *comCDE* coding sequence deleted and harboring *cat* gene, conferring chloramphenicol (Cm) resistance from pEVP3	This study
BASP2	D39Δ*comE* – D39 derivative with *comE* coding sequence deleted and harboring *cat* gene from pEVP3 (Cm resistant) as well as K56T point mutation in *rpsL* (Str resistant)	This study
BASP3	GA16833Δ*ftsK* – GA16833 derivative with *ftsK* coding sequence deleted in Tn*2009* and harboring *cat* gene from pEVP3, conferring chloramphenicol (Cm) resistance	This study
BASP4	D39Δ*comEA/EC* – D39 derivative with *comEA* and *comEC* coding sequences deleted and harboring *cat* gene from pEVP3 (Cm resistant) as well as K56T point mutation in *rpsL* (Str resistant)	This study
BASP5	TIGR4Δ*comE* – TIGR4 derivative with *comE* coding sequence deleted and harboring *cat* gene from pEVP3 (Cm resistant) as well as I100L point mutation in *folA* (Tmp resistant)	This study

BASP6	GA40410Δ*comE* – GA40410 derivative with *comE* coding sequence deleted and harboring *cat* gene from pEVP3 (Cm resistant) as well as I100L point mutation in *folA* (Tmp resistant)	This study
BASP7	GA43265Δ*comE* – GA43265 derivative with *comE* coding sequence deleted and harboring *cat* gene from pEVP3 (Cm resistant) as well as I100L point mutation in *folA* (Tmp resistant)	This study
BASP8	GA44194Δ*comCDE* – GA44194 derivative with *comCDE* coding sequence deleted and harboring *cat* gene, conferring chloramphenicol (Cm) resistance from pEVP3	This study
WT D39$^{Ery/Str}$	D39 derivative transformed with genomic DNA containing *ermB* (Ery resistant) and K56T point mutation in *rpsL* (Str resistant)	This study
GA47179	Clinical isolate from Georgia Emerging Infections Program, serotype 15A, truncated Tn*6002* (17.2 kb) conferring Tet resistance (*tetM*), Ery resistance (*ermB*)	(3)
GA44194	Clinical isolate from Georgia Emerging Infections Program, serotype 19A, truncated Tn*6002* (17.0 kb) conferring Tet resistance (*tetM*), Ery resistance (*ermB*)	(3)
GA49542	Clinical isolate from Georgia Emerging Infections Program, serotype 9V, ICE Tn*2009* conferring Tet resistance (*tetM*), Ery resistance (*mefE*/*mel* on Mega)	(3)
GA44288	Clinical isolate from Georgia Emerging Infections Program, serotype 19A, ICE Tn*2010* conferring Tet resistance (*tetM*), Ery resistance (*ermB* and *mefE*/*mel* on Mega)	(3)
GA40410	Clinical isolate from Georgia Emerging Infections Program, serotype 19A and conferring Tmp resistance	This study
GA43265	Clinical isolate from Georgia Emerging Infections Program, serotype 19A and conferring Tmp resistance	This study
Bacillus subtilis		
CMJ253	Tn*916*-containing strain, Tet resistant	(5)
LDW737	Strain with Tn*916* and Tn*916*-related ICE circular junction cloned into *amyE* gene and spectinomycin resistant	(6)
CAL419	Strain with *comK* coding sequence deleted and harboring *cat* gene (Cm resistant) as well as well streptomycin resistance	(5)

Table S2. Primers and probes used in this study.

Primer Name	Primer Sequence 5' → 3'	Reference or Source
**Conventional PCR**		
D39_recA_5F	TGAGCAGGCACGAAGCAAGAC	This study
D39_recA_3R	AGGAGCGACAAGAAACAGCAAACT	This study
BSA25	TTGCGGACTTAGGTTCTGTG	This study
BSA26	ACAAACCATGTCATTTGCGTAAAG	This study
EVP3_CmF	GGTATCGATAAGCTTGATGAAAA	This study
EVP3_CmR	TTAGTGACATTAGAAAACCGACTG	This study
BSA17	AATCAAGAATCAAAAGGTCGTTCCC	This study
BSA18	TTTTCATCAAGCTTATCGATACCGAAAAAGTCTCCTTTCTACCTAGCG	This study
BSA19.1	GTACTTTTTACAGTCGGTTTTCTAATGTCACTAAGTACAAATCGACAGGAAACAGTCAA	This study
BSA20	TCCCATACATTCCTTTTCCTCTTTG	This study
BSA17.1	GACCATCAAACATTCATTCAGCCA	This study
BSA20.1	CGATAGCTTTTAAAACTGCGGAAGA	This study
MS93	TAGTCAAAGCAAATCATAAATTGCG	This study
MS99	GCTTATCGATACCGTCGAATATTCTCTCTAGTCTCACTTGATGTTC	This study
MS100	CGGTTTTCTAATGTCACTAACTCTCAAAAGTGATTGACAATTAGC	This study
MS96	CATGCTCATCACAAAAGAGACGC	This study
MS101	GAACATCAAGTGAGACTAGAGAGAATATTCGACGGTATCGATAAGC	This study
MS102	GCTAATTGTCAATCACTTTTGAGAGTTAGTGACATTAGAAAACCG	This study
SL107	CAACATAGAAGACTCAGC	This study
SL108	GAATATCTAGAGTCAGAACC	This study
BSA1a	TCAGCTCCTTGCTTTTGATAGTCAG	This study
comEA_5RA3	TTTTCATCAAGCTTATCGATACCTCGTAAGAGGAAGAAAAAACAGTCG	This study

comEC_3FA3	CAGTCGGTTTTCTAATGTCACTAAGT GTTCGATAGGAAGGATAAATGTT	This study
BSA6a	ACACCGAGTACAGATGCAAATAAAA	This study
BSA11a	GCATTTGTTTCGATAAGGACACG	This study
SL115	CGGTTTTCTAATGTCACTAACTCTCA AAAGTGATTGACAATTAGC	This study
SL118	GCTTATCGATACCGTCGAGGAAAATT CCCAGCTTTGC	This study
SL119	CGCTATTTTGTCTGTTTGCCG	This study
SL116	GCTAATTGTCAATCACTTTTGAGAGT TAGTGACATTAGAAAACCG	This study
SL117	GCAAAGCTGGGAATTTTCCTCGACGG TATCGATAAGC	This study
SL199	CGCTATTTTGTCTGTTTGCCG	This study
**Quantitative PCR**		
Serotype 2_F	TGTTATCCCATATAAGAACCGAGTGT	(7)
Serotype 2_R	AAAATTACCCCAAAAGCTATCCAA	(7)
Serotype 2_probe	*HEX* – TTGCAATTTCAATTTTTTTGCCCCAAT CTC – *BHQ1*	(7)
Serotype 19F_F	TGAGGTTAAGATTGCTGATCG	(7)
Serotype 19F_R	CACGAATGAGAACTCGAATAAAAG	(7)
Serotype 19F_probe	*CY5* – CGCACTGTCAATTCACCTTC – *BHQ3*	(7)
tetM_qF1	AGGAAGCGTGGACAAAGGTA	This study
tetM_qR1	GAGTTTGTGCTTGTACGCCA	This study
int_qF1	ATTGCCACACATCACTCCAC	This study
int_qR1	CAAGACGCTCCTGTTGCTTC	This study
orf 20_qF1 (relaxase)	CAGCAGGTGGTCGAAAACAT	This study
orf 20_qR1 (relaxase)	ACCAGCTTCTTTGTTGTGCC	This study
ftsK_qF1	TCTCCCGGCACACTTCTTAA	This study
ftsK_qR1	TGGACGTTGACAAGCCAGTA	This study
cat_qF1	TCTCTGGTATTTGGACTCCTGT	This study
cat_qR1	TGCTGTAATAATGGGTAGAAGGT	This study
oLW526	AAACGTGAAGTATCTTCCTACAG	(6)

oLW527	TCGTCGTATCAAAGCTCATTC	(6)
ermB_qF1	TTTTGAAAGCCGTGCGTCTG	This study
ermB_qR1	CATCTGTGGTATGGCGGGTA	This study
comD_qF1	TCCGTGGTTTTCGACATGAT	This study
comD_qR1	ACTGAGCAACCAAACTTCGT	This study
comE_qF1	CCAGGTATCAGCCCTAGATTTTG	This study
comE_qR1	CGCAATTTATGAGATACCCCTGT	This study
Serotype 15A_F	AATTGCCTATAAACTCATTGAGATAG	(7)
Serotype 15A_R	CCATAGGAAGGAAATAGTATTTGTTC	(7)
Serotype 15A_probe	*FAM* – CCCGCAAACTCTGTCCT – *BHQ1*	(7)
Serotype 19A_F	CGCCTAGTCTAAATACCA	(7)
Serotype 19A_R	GAGGTCAACTATAATAGTAAGAG	(7)
Serotype 19A_probe	*FAM* – TATCAATGAGCCGATCCGTCACTT – *BHQ1*	(7)
Serotype 9V_F	AGGTATCCTATATACTGCTTTAGG	(7)
Serotype 9V_R	CGAATCTGCCAATATCTGAAAG	(7)
Serotype 9V_probe	*HEX* – ACACATTGACAACCGCT – *BHQ1*	(7)
q16s_F2	CCAGATGGACCTGCGTTGTAT	(8)
q16s_R2	TCCGTCCATTGCCGAAGATT	(8)

Table S3. Genetic identity and rF data for D39$^{Str/Tmp}$ and GA16833$^{Tet/Ery}$ or GA47281$^{Tet/Ery}$ bioreactor co-inoculation strains and recombinants.

**Bioreactor Co-Inoculation Strains**		
**Strain**	**MLST Sequence Type/Serotype**	
wt D39$^{Str/Tmp}$	ST 595/serotype 2	
wt GA16833$^{Tet/Ery}$ (Tn*2009*)	ST 236$_{slv}$/serotype 19F	
**Recombinants**		
**Recombinant Antibiotic Selection**	**MLST Sequence Type/Serotype**	**rF**
Tet+Str	ST 595/serotype 2	$2.60x10^{-4}±2.08x10^{-4}$
Ery+Str	ST 595/serotype 2	$1.37x10^{-5}±7.34x10^{-6}$
Tet+Ery+Str	ST 595/serotype 2	$2.00x10^{-5}±2.09x10^{-5}$
Ery+Str+Tmp	ST 595/serotype 2	$6.42x10^{-6}±4.89x10^{-6}$
**Bioreactor Co-Inoculation Strains**		
**Strain**	**MLST Sequence Type/Serotype**	
wt D39$^{Str/Tmp}$	ST 595/serotype 2	
wt GA47281$^{Tet/Ery}$ (Tn*2010*)	ST 3039/serotype 19F	
**Recombinants**		
**Recombinant Antibiotic Selection**	**MLST Sequence Type/Serotype**	**rF**
Tet+Str	ST 595/serotype 2	$1.34x10^{-4}±1.62x10^{-4}$
Ery+Str	ST 595/serotype 2	$1.63x10^{-4}±2.06x10^{-4}$
Tet+Ery+Str	ST 595/serotype 2	$1.39x10^{-4}±1.82x10^{-4}$
Ery+Str+Tmp	ST 595/serotype 2	$1.65x10^{-4}±1.93x10^{-4}$

Table S4. Homologous recombination of variably sized donor DNA fragments with intact ICE into bioreactor recombinant genomes.

**Donor (ICE, MLST)**	**Recipient (MLST)**	**Recombinant (MLST)**	**Left (5') of ICE (bp)**	**Right (3') of ICE (bp)**	**Size of Recombined Donor Fragment with ICE (bp)**
GA47281 (Tn*2010*$^{Tet/Ery}$, ST 3039)	D39$^{Str/Tmp}$ (ST 595)	Tet + Str (ST 595)	4890	7158	38,438
GA47281 (Tn*2010*$^{Tet/Ery}$, ST 3039)	D39$^{Str/Tmp}$ (ST 595)	Ery + Str (ST 595)	918	7158	34,466
GA47179 (partial Tn*6002*$^{Tet/Ery}$, ST 63)	D39^{Str} (ST 595)	Tet + Str (ST 595)	82345	1480	101,025
GA44288 (Tn*2010*$^{Tet/Ery}$, ST 320)	D39^{Str} (ST 595)	Tet + Str (ST 595)	4717	12274	43,381
GA44288 (Tn*2010*$^{Tet/Ery}$, ST 320)	D39^{Str} (ST 595)	Tet + Str (ST 595)	4718	4132	35,240
GA44288 (Tn*2010*$^{Tet/Ery}$, ST 320)	D39^{Str} (ST 595)	Tet + Str (ST 595)	539	17536	44,465
BASP1 (Tn*2009*$^{Tet/Ery}$, ST 236)	GA40410^{Tmp} (ST 1936)	Tet + Tmp (ST 1936)	14916	2042	40,499
BASP1 (Tn*2009*$^{Tet/Ery}$, ST 236)	GA40410^{Tmp} (ST 1936)	Tet + Tmp (ST 1936)	1088	49071	73,700
BASP1	GA40410^{Tmp} (ST 1936)	Tet + Tmp (ST 1936)	29167	39616	92,324

(Tn*2009*$^{Tet/Ery}$, ST 236)					
BASP1 (Tn*2009*$^{Tet/Ery}$, ST 236)	GA43265^{Tmp} (ST 2584)	Tet + Tmp (ST 2584)	44458	21649	89,648
BASP1 (Tn*2009*$^{Tet/Ery}$, ST 236)	GA43265^{Tmp} (ST 2584)	Tet + Tmp (ST 2584)	14577	12520	50,638

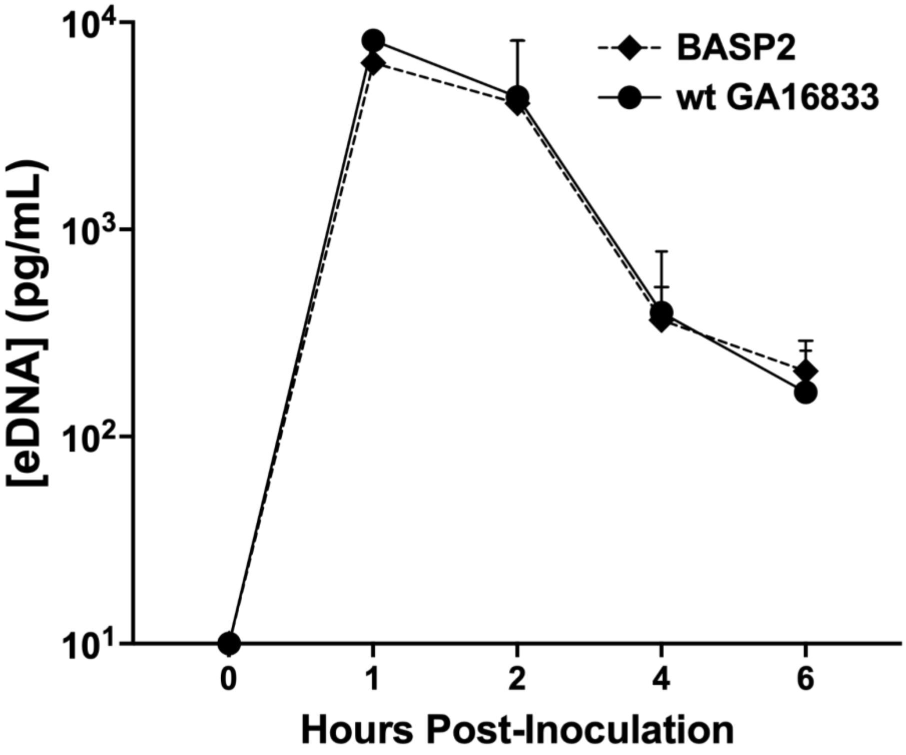

Figure S1. Similar extracellular DNA concentrations are secreted from competence-deficient mutant recipient strain BASP2 and Tn*2009* ICE donor GA16833 in nasopharyngeal dual-strain biofilms. Extracellular DNA from spent media of BASP2 (D39Δ*comE*) and GA16833$^{Tet/Ery}$ bioreactor co-inoculation was quantified with serotype-specific qPCR for serotypes 2 and 19F.

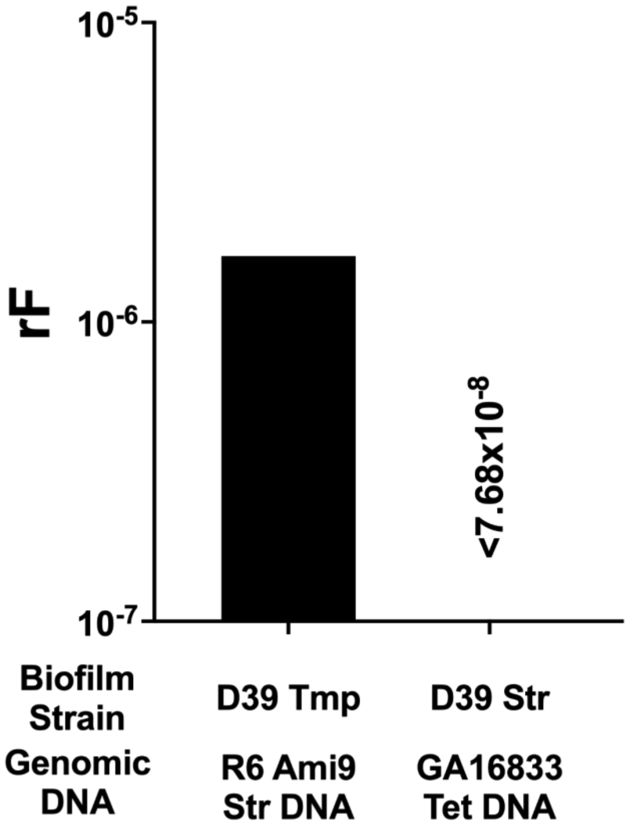

Figure S2. Single recipient strain biofilm transforms point mutation-mediated resistance but not Tn*916*-related ICE resistance. Recipient pneumococcal strains D39^{Tmp} or D39^{Str} were inoculated in a bioreactor at 35 °C on a confluent monolayer of human nasopharyngeal Detroit cells such that single strain biofilms formed. Flowing bioreactor media was supplemented with a final concentration of 700 ng/mL of genomic DNA from streptomycin (Str)-resistant R6 Ami9 or tetracycline (Tet)-resistant GA16833$^{\mathrm{Tn}\mathit{2009}}$. After a 6-hr total incubation, recombination frequencies per μg DNA for D39 uptake of Str or Tet resistance were calculated.

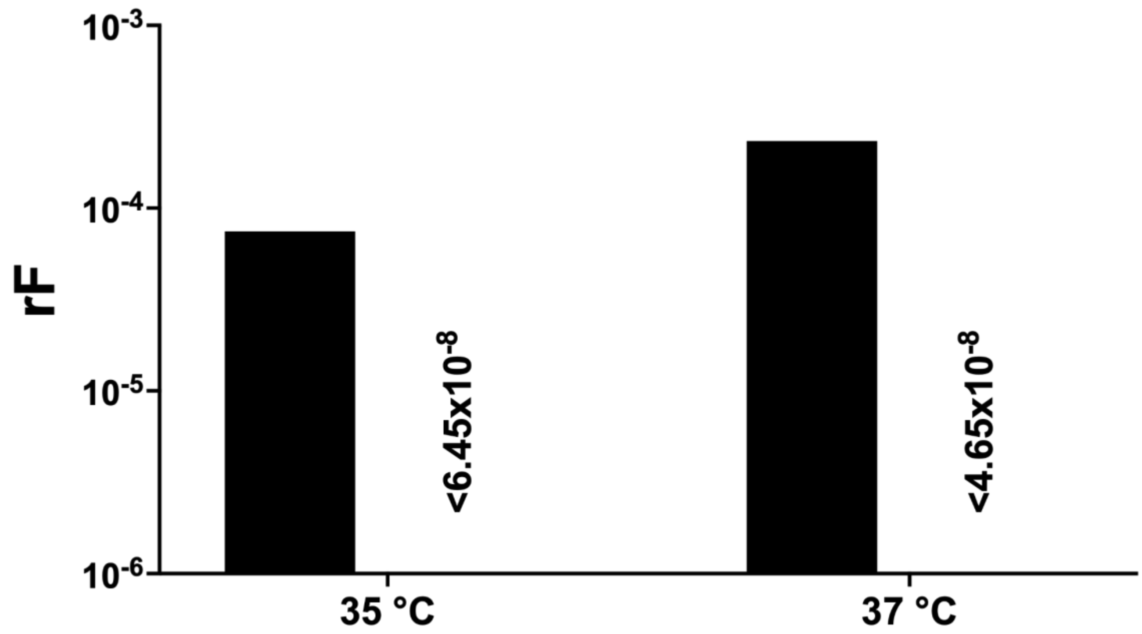

Figure S3. *In vitro* transformation at 35 °C and 37 °C facilitates recipient uptake of point mutation-mediated resistance but not Tn*916*-related ICE resistance. Pre-competent wildtype D39 cells were incubated in complete transformation medium with 100 ng/mL CSP1 and 500 ng of genomic DNA from R6 Ami9^{Str} or GA16833 Tn*2009*$^{Tet/Ery}$ at 35 °C or 37 °C. After a 2-hr total incubation time, recombination frequencies per μg DNA were calculated for D39 uptake of Str (black bars) or Tet resistance (< numerical values).

SUPPLEMENTAL MATERIAL BIBLIOGRAPHY AND REFERENCES CITED

1. Avery OT, Macleod CM, McCarty M. Studies on the Chemical Nature of the Substance Inducing Transformation of Pneumococcal Types : Induction of Transformation by a Desoxyribonucleic Acid Fraction Isolated from Pneumococcus Type Iii. J Exp Med. 1944;79(2):137-58. Epub 1944/02/01. doi: 10.1084/jem.79.2.137. PubMed PMID: 19871359; PMCID: PMC2135445.

2. Tettelin H, Nelson KE, Paulsen IT, Eisen JA, Read TD, Peterson S, Heidelberg J, DeBoy RT, Haft DH, Dodson RJ, Durkin AS, Gwinn M, Kolonay JF, Nelson WC, Peterson JD, Umayam LA, White O, Salzberg SL, Lewis MR, Radune D, Holtzapple E, Khouri H, Wolf AM, Utterback TR, Hansen CL, McDonald LA, Feldblyum TV, Angiuoli S, Dickinson T, Hickey EK, Holt IE, Loftus BJ, Yang F, Smith HO, Venter JC, Dougherty BA, Morrison DA, Hollingshead SK, Fraser CM. Complete genome sequence of a virulent isolate of Streptococcus pneumoniae. Science. 2001;293(5529):498-506. Epub 2001/07/21. doi: 10.1126/science.1061217. PubMed PMID: 11463916.

3. Chancey ST, Agrawal S, Schroeder MR, Farley MM, Tettelin H, Stephens DS. Composite mobile genetic elements disseminating macrolide resistance in Streptococcus pneumoniae. Front Microbiol. 2015;6:26. Epub 2015/02/25. doi: 10.3389/fmicb.2015.00026. PubMed PMID: 25709602; PMCID: PMC4321634.

4. Lattar SM, Wu X, Brophy J, Sakai F, Klugman KP, Vidal JE. A Mechanism of Unidirectional Transformation, Leading to Antibiotic Resistance, Occurs within Nasopharyngeal Pneumococcal Biofilm Consortia. MBio. 2018;9(3). Epub 2018/05/17. doi: 10.1128/mBio.00561-18. PubMed PMID: 29764945; PMCID: PMC5954218.

5. Johnson CM, Grossman AD. Identification of host genes that affect acquisition of an integrative and conjugative element in Bacillus subtilis. Mol Microbiol. 2014;93(6):1284-301. Epub 2014/07/30. doi: 10.1111/mmi.12736. PubMed PMID: 25069588; PMCID: PMC4160349.

6. Wright LD, Grossman AD. Autonomous Replication of the Conjugative Transposon Tn916. J Bacteriol. 2016;198(24):3355-66. Epub 2016/10/05. doi: 10.1128/JB.00639-16. PubMed PMID: 27698087; PMCID: PMC5116939.

7. Pimenta FC, Roundtree A, Soysal A, Bakir M, du Plessis M, Wolter N, von Gottberg A, McGee L, Carvalho Mda G, Beall B. Sequential triplex real-time PCR assay for detecting 21 pneumococcal capsular serotypes that account for a high global disease burden. J Clin Microbiol. 2013;51(2):647-52. Epub 2012/12/12. doi: 10.1128/JCM.02927-12. PubMed PMID: 23224094; PMCID: PMC3553924.

8. Schroeder MR, Lohsen S, Chancey ST, Stephens DS. High-Level Macrolide Resistance Due to the Mega Element [mef(E)/mel] in Streptococcus pneumoniae. Front Microbiol. 2019;10:868. Epub 2019/05/21. doi: 10.3389/fmicb.2019.00868. PubMed PMID: 31105666; PMCID: PMC6491947.
